# Augmented Reality, Mixed Reality, Computer Vision, and Three-Dimensional Modeling in Fertility-Preserving Minimally Invasive Gynecologic Surgery: A Scoping Review with Narrative Synthesis

**DOI:** 10.3390/medicina62091790

**Published:** 2026-09-17

**Authors:** Eleni Karatrasoglou, Alexandros Rodolakis, Themistoklis Grigoriadis, Athanasios Protopapas

**Affiliations:** 1 Department of Gynecology, National and Kapodistrian University of Athens, Mikras Asias 75, 11527 Athens, Greece; 2 Department of Minimally Invasive Gynecologic Surgery, Alexandra University Hospital, 11528 Athens, Greece

**Keywords:** augmented reality, mixed reality, computer vision, artificial intelligence, three-dimensional modeling, minimally invasive gynecologic surgery, reproductive surgery, myomectomy, adenomyomectomy, adenomyosis, endometriosis, fertility-preserving surgery

## Abstract

*Background and Objectives*: Augmented reality (AR), mixed reality (MR), computer vision, artificial intelligence (AI), and three-dimensional (3D) modeling may be particularly relevant in fertility-preserving gynecologic surgery, where disease must be treated while uterine architecture, reproductive anatomy, and future fertility potential are preserved. This scoping review mapped and appraised the current evidence, with emphasis on myomectomy, adenomyomectomy, and endometriosis surgery. *Materials and Methods*: A scoping review with narrative synthesis was conducted and reported according to the PRISMA Extension for Scoping Reviews (PRISMA-ScR). PubMed/MEDLINE, Scopus, and Google Scholar were searched from database inception to 16 May 2026. Records were appraised with design-appropriate instruments: Joanna Briggs Institute (JBI) principles, Risk Of Bias In Non-randomized Studies of Interventions (ROBINS-I), randomized-trial risk-of-bias domains, and structured feasibility criteria. *Results*: Of 528 records identified, 20 were included. Eleven involved application of the technology in the care of patients; of these, only two were comparative—one retrospective matched case–control study of AR-assisted myomectomy and adenomyomectomy, and one single-center randomized controlled trial of 3D-printed model-assisted myomectomy. The remaining nine were enabling technologies validated on image datasets, operative video, ex vivo models, or animal models. Only one study reported a reproductive outcome—pregnancy rates in a retrospective matched series of 34 patients, which did not differ between groups—and no study reported live birth, obstetric, or long-term reproductive-safety outcomes. *Conclusions*: Current evidence supports technical feasibility and preoperative planning value but does not demonstrate improvement in reproductive outcomes; here “fertility-preserving” denotes the clinical context of the surgery rather than a demonstrated reproductive benefit. Adequately powered controlled studies reporting fertility-relevant endpoints are required.

## 1. Introduction

Minimally invasive gynecologic surgery has transformed the management of benign gynecologic disease in reproductive-age women. Compared with open surgery, laparoscopic and ultra-minimally invasive approaches are associated with reduced tissue trauma, shorter hospitalization, lower postoperative pain, faster recovery, and improved perioperative outcomes in appropriately selected patients [1,2]. These advantages are particularly important in reproductive-age patients, in whom preservation of uterine integrity, pelvic anatomy, ovarian reserve, and future fertility potential is central to surgical decision-making.

Despite these benefits, complex fertility-preserving pelvic surgery remains technically demanding. In many procedures, pathology is partly or completely hidden beneath the visible surface, normal tissue planes may be distorted, and critical structures must be preserved in an operative field with limited tactile feedback. This challenge is especially important in laparoscopic myomectomy, adenomyomectomy, and deep endometriosis surgery, where the surgical objective is not simply disease removal but disease treatment with preservation of reproductive function.

In laparoscopic myomectomy, operative difficulty depends on fibroid size, number, depth, location, and degree of protrusion from the uterine surface. Deep intramural, posterior, broad-ligament, or poorly protruding myomas may be difficult to localize precisely during laparoscopy, particularly when the external uterine contour provides limited guidance [3,4,5]. In fertility-preserving adenomyomectomy, the difficulty is different. Adenomyotic lesions are often poorly circumscribed, infiltrative, and close to the endometrial cavity, creating a surgical balance between adequate excision and preservation of healthy myometrium [6]. In deep endometriosis, the main challenge is frequently distorted anatomy, fibrosis, adhesions, and the close relationship of disease to the ureters, bowel, bladder, uterosacral ligaments, rectovaginal septum, and pelvic sidewall [7,8,9,10,11].

Against this background, augmented reality (AR), mixed reality (MR), computer vision, artificial intelligence (AI), and three-dimensional (3D) modeling have emerged as potentially useful adjuncts. AR generally refers to the overlay of digital information onto the real operative view, while MR allows interaction with digital models in relation to the physical environment. Computer vision and AI may support automated organ or lesion recognition, segmentation, tracking, and registration. Three-dimensional modeling and virtual-reality visualization may support preoperative planning, anatomical communication, and surgical rehearsal [12,13,14]. For the operating surgeon these technologies address four distinct tasks, and it is worth stating them separately because they sit at very different levels of validation. First, preoperative 3D and virtual-reality models permit surgical rehearsal: the surgeon can simulate the planned incision, assess resection margins, and map the approach to deep or multiple lesions before touching tissue. Second, intraoperative AR and MR provide guidance during the procedure itself, projecting subsurface structures—hidden lesions, lesion margins, the endometrial cavity, and complex vasculature—onto or alongside the live operative view. Third, computer vision algorithms track instruments and anatomical landmarks and can update the displayed model to compensate for soft-tissue deformation during laparoscopic or robotic dissection. Fourth, AI systems monitoring the operative field may in principle deliver context-aware feedback through difficult tissue planes. The mechanistic rationale in each case is the same: better anatomical information should support more selective excision, less unnecessary dissection, and better preservation of functional tissue. It is important to be explicit that this is a rationale and not a demonstrated effect. As set out in Section 3.7 and Section 4, no study identified in this review quantifies a reduction in intraoperative complications attributable to any of these technologies, and no specific magnitude of benefit should be inferred from the plausibility of the mechanism.

A central issue in this field is terminology. True intraoperative AR overlay, MR visualization, computer vision-based registration, AI lesion recognition, and 3D surgical planning are related technologies, but they are not equivalent. A real-time registered AR overlay during laparoscopy is not the same as preoperative 3D modeling, a holographic MR display, AI-based lesion recognition, or a 3D-printed surgical planning model. This distinction is particularly important in reproductive surgery, where claims of improved precision, reduced tissue trauma, lower complication risk, or better fertility outcomes require direct supporting evidence.

The aim of this scoping review was to map and critically appraise the current evidence regarding AR, MR, computer vision, and 3D modeling in fertility-preserving minimally invasive gynecologic surgery, with emphasis on myomectomy, adenomyomectomy, adenomyosis surgery, and endometriosis surgery. A further aim was to define the maturity of the evidence, identify major gaps, and clarify future research priorities.

## 2. Materials and Methods

### 2.1. Study Design

**Figure 1:**
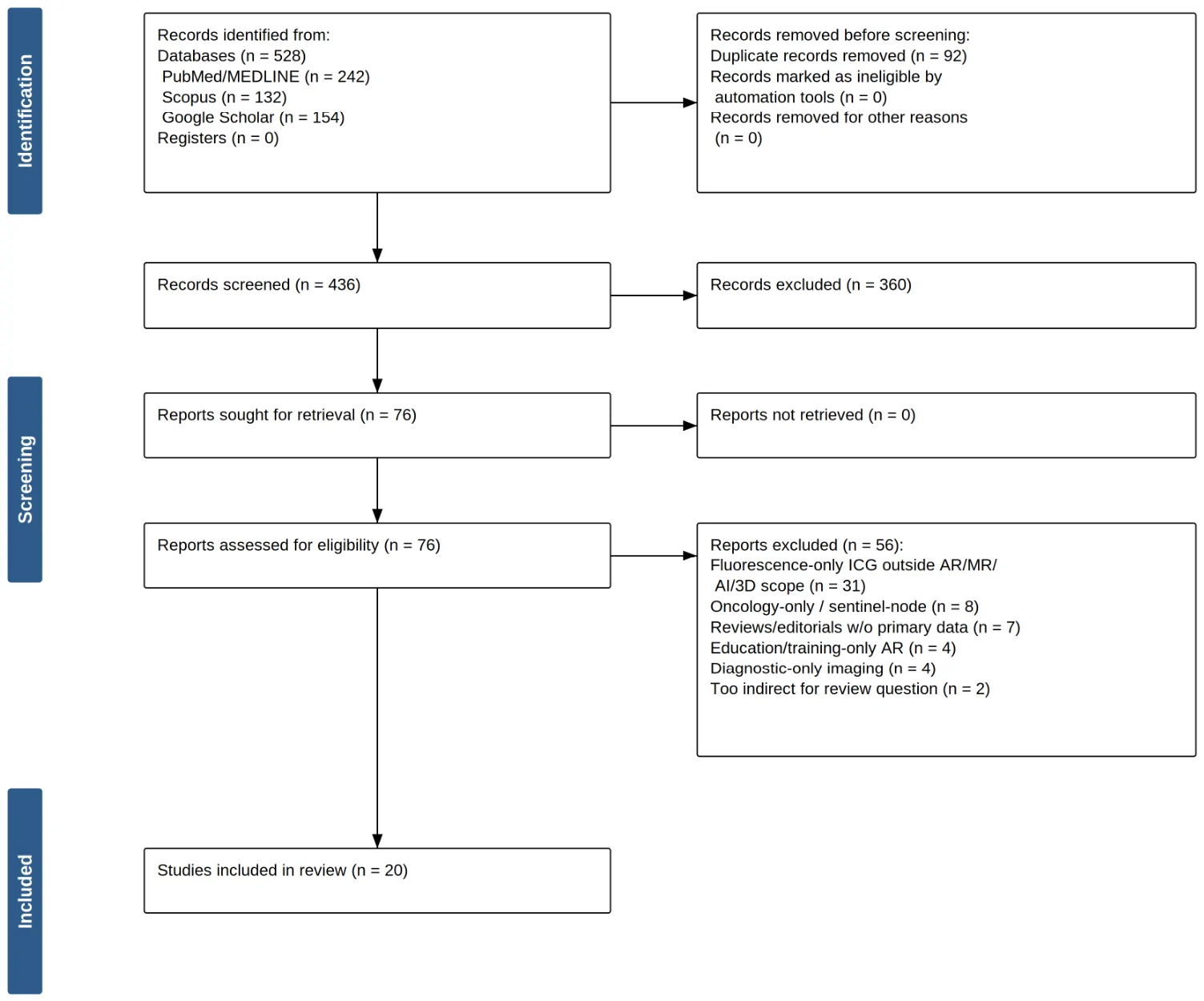
PRISMA 2020 flow diagram of study identification, screening, eligibility assessment, and inclusion of studies evaluating augmented reality, mixed reality, computer vision/artificial intelligence, and three-dimensional modeling in fertility-preserving minimally invasive gynecologic surgery. No meta-analysis was performed because of heterogeneity in study design, technology type, procedures, and outcomes.

This study was designed as a scoping review with narrative synthesis. A scoping design was selected because the available literature is heterogeneous, early-phase, and unsuitable for meta-analysis. This scoping review was conducted and is reported in accordance with the Preferred Reporting Items for Systematic Reviews and Meta-Analyses extension for Scoping Reviews (PRISMA-ScR), the reporting guideline specifically developed for scoping reviews [15], together with PRISMA 2020 reporting principles where applicable [16]. A completed PRISMA-ScR checklist is provided as Supplementary Material (Table S1), and the identification and selection of sources of evidence are documented in the PRISMA flow diagram (Figure 1). The review was not registered in a public registry, and no separate review protocol was published. No prospective registration was undertaken because PROSPERO does not accept scoping review protocols, and no alternative registry was in routine institutional use for this review type at the time the review was initiated; the absence of registration is acknowledged explicitly as a limitation (Section 4). The review methods were nonetheless defined a priori, before the formal search was executed, and comprised the population–concept–context framework (Section 2.2), the inclusion and exclusion criteria (Section 2.4 and Section 2.5), the technology classification scheme (Section 2.8), the data-charting variables (Section 2.7), and the appraisal approach (Section 2.9 and Section 2.10). One post hoc refinement was made and is reported transparently: during revision, the scope was narrowed to AR, MR, computer vision and AI, and 3D modeling, which led to the exclusion of fluorescence-only indocyanine green (ICG) records at the full-text stage (Section 2.5). No other eligibility criterion was modified after the search. The complete search strategies as executed in each source are provided as Supplementary Material (Table S4).

### 2.2. Eligibility Framework

Eligibility was structured according to a population, concept, and context framework. The population consisted of patients undergoing minimally invasive gynecologic surgery, with emphasis on reproductive-age women and fertility-preserving procedures. The concept was the use of AR, MR, computer vision, AI-enabled visual recognition, 3D modeling, 3D printing, or virtual-reality visualization intended to improve anatomical orientation, lesion localization, surgical planning, registration, tracking, or operative guidance. The context was benign minimally invasive gynecologic surgery, with core relevance to myomectomy, adenomyomectomy, adenomyosis surgery, endometriosis surgery, and anatomy-preserving reproductive pelvic surgery.

### 2.3. Information Sources and Search Strategy

The final reproducible literature search was performed in PubMed/MEDLINE, Scopus, and Google Scholar from database inception to 16 May 2026. The primary literature search was executed on 11 February 2026, and all screening, full-text assessment, data charting, and appraisal were based on that search. A confirmatory re-run of the identical search strategies was performed on 16 May 2026, immediately before submission, in order to verify that no newly indexed eligible record had appeared; this confirmatory search retrieved no additional eligible record, and the included evidence base was therefore unchanged. The record counts reported in Figure 1 correspond to the 11 February 2026 search.

The same concept blocks were applied to all three sources and combined with AND: a visualization and computing technology block (augmented reality, mixed reality, image-guided and computer-assisted surgery, computer vision, artificial intelligence, deep learning, three-dimensional modeling, and indocyanine green), a gynecologic procedure block (gynecologic surgery, gynecology, laparoscopy, and robotic surgery), and a benign uterine and endometriotic pathology block (myomectomy, myoma, fibroid, leiomyoma, adenomyosis, adenomyomectomy, endometriosis, deep infiltrating endometriosis, ureteric involvement, and fertility-preserving or reproductive surgery). Each string was rewritten to the field-tagging conventions of the platform concerned, because an identical literal string would have been syntactically invalid on at least two of the three platforms. PubMed/MEDLINE was searched with [Title/Abstract] tags and truncation, with animal-only records excluded by MeSH, limited to English-language human studies with preprints excluded, and retrieved 242 records. Scopus was searched with the TITLE-ABS-KEY field code, limited to English-language records with conference papers, books, book chapters, letters, editorials, notes, errata, and short surveys excluded where applicable, and retrieved 132 records. Google Scholar was searched with seven bounded phrase-pair queries in a private browser session, screening up to the first 25 relevance-ranked results per query, and 154 records were screened. The complete strategies as executed, with every search term, the interface limiters applied, and the yield of each source, are reproduced verbatim in Supplementary Table S4.

Because relevance-ranked Google Scholar output cannot be reproduced exactly, this source was used as a supplementary coverage safeguard rather than as a primary retrieval channel. On cross-checking the final included set against the source of first retrieval, Google Scholar contributed no unique included record: every one of the 20 included studies was independently retrieved by the PubMed/MEDLINE or Scopus searches, both of which are fully reproducible. The non-reproducibility of the Google Scholar ranking therefore does not affect the composition of the evidence base, although it is acknowledged as a limitation (Section 4).

Reference lists of included articles and relevant reviews were checked, but no additional unique record was added through citation searching.

### 2.4. Inclusion Criteria

Records were included if they met at least one of the following criteria: (1) clinical or technical use of intraoperative AR in minimally invasive gynecologic surgery; (2) MR visualization relevant to myomectomy, adenomyomectomy, or benign gynecologic surgical planning; (3) computer vision or AI methods relevant to gynecologic laparoscopy, uterus detection, AR automation, or endometriosis lesion recognition; (4) 3D modeling, 3D printing, or virtual-reality visualization directly relevant to benign gynecologic surgical planning, myomectomy, adenomyomectomy, or endometriosis surgery; or (5) experimental, ex vivo, animal-model, dataset, or technical studies if they directly informed AR, MR, AI, computer vision, and 3D modeling workflows for benign gynecologic surgery.

### 2.5. Exclusion Criteria

Records were excluded if they focused exclusively on fluorescence-only indocyanine green imaging, gynecologic oncology, sentinel lymph node mapping, non-gynecologic surgery, diagnostic-only imaging without surgical planning relevance, general robotic surgery without AR, MR, AI, computer vision, or 3D modeling components, medical education-only AR, conference abstracts without sufficient procedural detail, books or book chapters, preprints, editorials, or reviews without usable primary technical or clinical data.

Fluorescence-only indocyanine green studies were excluded from the core evidence synthesis after full-text review because the revised scope was narrowed to AR, MR, computer vision and AI, and 3D modeling. Gynecologic oncology-only and sentinel-node studies were excluded because their operative goals differ from benign fertility-preserving surgery. The presence of the term “indocyanine green” within the search strings is deliberate and is not inconsistent with this exclusion. ICG terminology was retained in the search in order to retrieve, rather than to exclude, records in which fluorescence imaging was combined with AR overlay, computer vision, or 3D navigation, since such hybrid records are eligible. Records were excluded only where fluorescence was the sole visualization modality and no AR, MR, computer vision and AI, or 3D modeling component was present. Exclusion was thus applied at the full-text stage on the basis of study content, not at the search stage on the basis of terminology. Mandatory eligibility parameters applied at full text. To make the eligibility decision reproducible rather than a matter of reviewer judgment, four parameters were applied to every retrieved full text, each framed as a question answerable from the record itself. P1, population: does the record report a procedure performed on the female reproductive tract, and is that procedure uterus-preserving or otherwise anatomy-preserving? For enabling-technology records (Stream B, Section 3.2), P1 is satisfied where the anatomical target, the imaging source, or the operative setting is gynecologic, irrespective of whether the index procedure was itself uterus-preserving; a computer vision development study conducted during total laparoscopic hysterectomy [17] therefore satisfies P1, whereas a record reporting a clinical outcome of hysterectomy would not. P2, technology: is the AR, MR, computer vision, AI, or 3D modeling component described in sufficient detail to be assigned to a category in Table 1, and is it applied or evaluated within the record rather than merely named? P3, context: is the technology applied to surgical planning, intraoperative guidance, intraoperative orientation or navigation, or operative decision-making, rather than exclusively to diagnosis, education, training, patient counseling, or postoperative assessment? P4, primary data: does the record report at least one primary clinical, technical, dataset, or experimental result attributable to that record? A record failing any single parameter was excluded. These parameters were applied to every retrieved full text and are not specific to any one exclusion category: the categories listed in Supplementary Table S2 are the recurring modes of failure, with oncology-only records failing P1, education-only and diagnostic-only records failing P3, and reviews without primary data failing P4, and each record was assigned to the most specific applicable category.

### 2.6. Study Selection

Titles and abstracts were screened for relevance, followed by full-text review of potentially eligible records. Screening and data charting were not performed independently and in duplicate. The first author screened all titles and abstracts, retrieved and assessed all full texts, and charted all data; the senior author subsequently verified the eligibility decisions and the charted data against the source articles. Disagreements regarding eligibility, technology category, or evidence classification were resolved by consensus. Because the senior author reviewed the first author’s decisions rather than screening the records independently, no inter-reviewer agreement statistic (e.g., Cohen’s κ) could be computed, and none is reported. This single-screener design with senior verification is acknowledged as a limitation (Section 4). Reasons for exclusion at the full-text stage were documented prospectively at the level of exclusion category rather than at the level of the individual record, and a record-level bibliographic log of the 56 excluded full texts was not retained. The operational criterion applied within each exclusion category, together with the corresponding counts, is therefore set out in full in Supplementary Material (Table S2), and the category totals are reported in Figure 1. This limitation of the audit trail is stated explicitly in Section 4.

During revision, all included records were cross-checked against the evidence tables to ensure that every record counted in the qualitative synthesis was assigned to a technology category as defined in Table 1 and was represented in Table 2. The complete record-to-table correspondence, showing for each of the 20 included records its technology category, its evidence stream, and every table in which it appears, is set out in Supplementary Table S3, Panel C.

### 2.7. Data Extraction

For each included record, the following variables were extracted where available: first author, year, country or setting, study design, technology category, procedure or disease focus, sample size, imaging modality or platform, intended role of the technology, reported perioperative outcomes, complications, fertility or pregnancy outcomes, evidence level, and main limitations.

Data not reported in the source were recorded as not reported (NR). Data not applicable to a study design were recorded as not applicable (NA).

### 2.8. Technology Classification

To avoid treating all technologies as equivalent to AR, included studies were categorized as: (1) true intraoperative AR overlay; (2) MR visualization; (3) AI and computer vision enabling technologies; and (4) preoperative 3D modeling, 3D printing, or virtual-reality surgical planning. These categories are defined in Table 1 and applied to all included studies in Table 2.

### 2.9. Quality and Applicability Appraisal

Because the included studies varied widely in design, a single risk-of-bias tool was not appropriate for all records. Case reports and case series were appraised according to JBI critical appraisal principles [28]. The retrospective matched case–control study was assessed according to ROBINS-I domains [29]. The randomized trial was assessed using standard randomized-trial risk-of-bias domains, including randomization, allocation, blinding limitations, missing data, and outcome reporting. Technical, dataset, computer vision, and feasibility studies were assessed using a structured feasibility appraisal framework considering study design, sample size, directness of evidence, validation method, outcome reporting, follow-up, workflow assessment, and major bias concerns.

The appraisal was not used to exclude studies but to interpret the maturity and applicability of the evidence.

### 2.10. Evidence Level Classification

Approximate evidence levels were assigned using a simplified Oxford Centre for Evidence-Based Medicine hierarchy [30]. The levels are defined below in descending order of internal validity. Level I: systematic review or meta-analysis of randomized controlled trials, or an individual randomized trial with narrow confidence intervals; no included record met this level, and Level I therefore does not appear in any table. Level II: individual randomized controlled trial. Level III: non-randomized comparative study with a concurrent or matched comparator, including prospective or retrospective cohort and matched case–control designs. Level IV: single-arm cohort study, prospective or retrospective case series with more than one participant, feasibility study in patients, or structured surgeon-assessment study without a comparator. Level V: individual case report, technical development report, dataset or annotation study, bench or ex vivo experimental study, animal-model study, or mechanism-based reasoning. Where a record combined features of two adjacent levels, the lower (less internally valid) level was assigned, and no composite designation such as “IV–V” is used. These levels describe study design and the directness of the evidence to the review question; they are not certainty-of-evidence grades in the GRADE sense, and no certainty rating is claimed. The levels were not used to weight, rank, or exclude records, and a higher level does not by itself indicate that a technology is clinically effective; the Level II randomized trial in this review, for example, reports no fertility outcome. This definition is reproduced as a footnote beneath every table in which an evidence level is reported.

## 3. Results

### 3.1. Study Selection

The search identified 528 records, including 242 from PubMed/MEDLINE, 132 from Scopus, and 154 from Google Scholar. Citation searching did not identify additional unique records. After removal of 92 duplicate records, 436 records underwent title and abstract screening. Of these, 360 were excluded. Seventy-six reports were sought for retrieval, and all were retrieved or had sufficient publisher abstract information for eligibility assessment. Seventy-six full-text reports were assessed for eligibility. Fifty-six reports were excluded after full-text assessment: fluorescence-only indocyanine green image-guidance adjuncts outside the narrowed AR, MR, AI, computer vision, and 3D modeling scope (n = 31), oncology-only or sentinel-node navigation studies without benign fertility-preserving relevance (n = 8), reviews or editorials without usable primary technical or clinical data (n = 7), education/training-only AR or simulation studies (n = 4), diagnostic-only or non-surgical imaging studies (n = 4), and studies considered too indirect for the review question (n = 2).

Twenty records were included in the qualitative synthesis. No meta-analysis was performed because of heterogeneity in study designs, technologies, procedures, and outcomes. The PRISMA 2020 flow diagram is shown in Figure 1.

### 3.2. General Overview of the Included Evidence

The 20 included records were heterogeneous in technology type, clinical setting, and evidence maturity. They comprised true intraoperative AR overlay studies, MR applications, AI and computer vision studies, and 3D modeling or 3D printing studies. The operational categories used in this review are shown in Table 1, and all included records are mapped in Table 2.

No study demonstrated improved live birth rate, pregnancy rate, uterine rupture risk, obstetric outcome, or long-term fertility outcome. Most AR and MR evidence remained feasibility-based. The strongest comparative clinical evidence was found in 3D model-assisted myomectomy, particularly the randomized controlled trial by Li et al. [27]. Direct AR evidence was strongest in myomectomy and adenomyomectomy but was limited by small sample sizes and early-phase designs [3,4,5,6,18,21,22]. Endometriosis-related evidence was concentrated in 3D planning and AI lesion-recognition studies rather than real-time AR navigation [7,8,9,10,11]. Two levels of clinical maturity. Because the included records span designs from bench experiments to a randomized trial, the synthesis distinguishes throughout between two evidence streams, defined by a single criterion: whether the technology has been applied in the care of actual patients. Stream A—technologies applied in patients (n = 11). The technology was deployed intraoperatively, or its output was used in the preoperative planning of a real scheduled procedure: references [4,5,6,9,10,11,19,21,23,26,27]. Within this stream only two records are comparative—the retrospective matched case–control study of AR-assisted myomectomy and adenomyomectomy [21] and the randomized controlled trial of 3D-printed model-assisted single-port myomectomy [27]—and the remaining nine are case reports, small case series, or uncontrolled planning-evaluation studies. Reproductive outcomes are almost entirely absent from this stream. Exactly one record reports any reproductive outcome: in the matched AR series, 6 of the 11 AR patients who expressed a desire for pregnancy conceived, compared with 5 of the 12 such patients among matched controls (*p* = 1.00) [21]. No record in either stream reports live birth, miscarriage, uterine rupture, postoperative uterine integrity, or any obstetric or long-term reproductive-safety outcome, and no record demonstrates an improvement in any reproductive outcome attributable to the technology under study. Stream B—enabling technologies not yet applied in patient care (n = 9). The technology was developed or validated on image datasets, retrospective operative video, bench or ex vivo models, or animal models, without deployment in the care of a patient: references [3,7,8,17,18,20,22,24,25]. These records address genuine technical prerequisites for intraoperative AR—markerless registration, deformable tissue tracking, uterine contour detection, and automated lesion recognition—but they generate no clinical outcome evidence, and their presence should not be read as evidence that the corresponding clinical capability has been established. This distinction is applied consistently for a specific reason: the two streams sit at entirely different levels of clinical maturity, and presenting them together, as much of the existing narrative literature does, systematically overstates how far the field has advanced. Every record is assigned to a stream in Table 2; each results subsection below states which stream or streams it addresses; and the stream assignment is reproduced alongside the study-level appraisal in Supplementary Table S3.

### 3.3. Augmented Reality in Myomectomy and Adenomyomectomy

This subsection addresses both streams and identifies each record accordingly: of the six records discussed, four were applied in patients ([4,5,6,21]; Stream A) and two were experimental or animal-model studies ([3,18]; Stream B). The earliest AR evidence in myomectomy was reported by Bourdel et al., who evaluated AR-assisted myoma localization in an experimental uterine model [3]. In that study, AR improved localization accuracy compared with MRI-only localization, although the setting was experimental and did not provide patient-outcome evidence. The same group subsequently reported clinical AR use in three patients undergoing laparoscopic myomectomy, using MRI-derived 3D models of the uterus and myomas fused with laparoscopic video in real time [4]. These studies are summarized in Table 2 and Table 3.

**Table 3:** Myomectomy and Adenomyomectomy Evidence.

Study	Procedure	Technology	Design	Sample	Key Findings	Complications	Fertility Outcomes	Evidence Level
Bourdel et al., 2017 [3]	Myomectomy model	AR	Experimental user study	10 residents	Accuracy improved with AR versus MRI-only localization	NA	NA	V
Bourdel et al., 2017 [4]	Laparoscopic myomectomy	AR	Feasibility report	3	Real-time MRI-based myoma localization feasible	Not emphasized	NR	V
Bourdel et al., 2019 [6]	Adenomyomectomy	AR	Case report	2	Adenomyoma and cavity localized using AR	NR	NR	V
Chauvet et al., 2020 [5]	Laparoscopic myomectomy	AR + DTI	Case reports	2	Fiber orientation visualized to support incision planning	NR	NR	V
Ochi et al., 2023 [19]	Laparoscopic myomectomy	MR	Case report	1	Holographic model supported fibroid localization and endometrial awareness	Uneventful course	NR	V
Torabinia et al., 2022 [20]	Mock laparoscopic myomectomy	MR	Ex vivo feasibility	1 model	MR visualization perceived as useful	NA	NA	V
Comptour et al., 2025 [21]	Myomectomy/adenomyomectomy	AR	Retrospective matched case–control	34	Operative time non-inferior; no added adverse events	None reported	Pregnancy 6/11 with pregnancy desire vs. 5/12 in controls (*p* = 1.00); not a stated endpoint	III
Flaxman et al., 2024 [26]	Multifibroid uterus	3D printing	Planning evaluation	7 cases	Model review changed dissection route in 8/15 surgeon–case responses (5 surgeons across 7 cases; see Section 3.6)	NR	NR	IV
Li et al., 2026 [27]	Single-port laparoscopic multiple myomectomy	3D printing	Randomized controlled trial	133 randomized and analyzed (intention to treat); 110 per protocol (55/group)	Reduced operative time and surgeon workload	Similar between groups	NR	II

Abbreviations: AR, augmented reality; DTI, diffusion tensor imaging; MR, mixed reality; NA, not applicable; NR, not reported. Relationship to other tables: Table 3 is a procedure-focused subset of Table 2 and contains no record that does not also appear in Table 2. It is retained because it aligns the myomectomy and adenomyomectomy records with their complication and fertility-outcome reporting side by side, which Table 2 does not display; Table 4 is the corresponding subset for endometriosis-related and enabling computer vision records. The complete correspondence between the 20 included records and Table 2, Table 3 and Table 4 is given in Supplementary Table S3, Panel C. Evidence levels (defined in full in Section 2.10): II, individual randomized controlled trial; III, non-randomized comparative study with a concurrent or matched comparator; IV, single-arm cohort, case series, feasibility study in patients, or structured surgeon-assessment study without a comparator; V, case report, technical development report, dataset or annotation study, bench or ex vivo study, animal-model study, or mechanism-based reasoning. Level I (systematic review or meta-analysis of randomized trials) was not met by any included record. Levels describe study design and directness of evidence to the review question; they are not certainty-of-evidence grades and were not used to weight or exclude records.

**Table 4:** Endometriosis, Computer Vision, and 3D Planning Evidence.

Study	Context	Technology	Design	Sample	Main Contribution	Limitation
Netter et al., 2025 [7]	Endometriosis laparoscopy	AI lesion recognition	Multicenter proof-of-concept	112 videos	YOLOv5 detected 9 lesion classes with variable performance	Not real-time AR
Leibetseder et al., 2022 [8]	Endometriosis laparoscopy	AI detection/localization	Technical study	Video dataset	Faster R-CNN and Mask R-CNN for lesion localization	Moderate performance
Martel et al., 2026 [9]	Colorectal endometriosis	3D/VR modeling	Feasibility/expert evaluation	14 models	3D models perceived as useful for planning	No comparative outcomes
Borghese et al., 2022 [10]	Rectosigmoid endometriosis	3D virtual modeling	Prospective pilot cohort	7 women	High subjective correlation with operative findings	Small sample
Zhang et al., 2026 [11]	Deep endometriosis	MRI-based 3D modeling	Feasibility/survey/preliminary prospective	NR	Supported planning and anatomical understanding	No real-time AR
Sato et al., 2019 [17]	Gynecologic laparoscopy	Computer vision	Preliminary video study	19 cases	Explored panoramic reconstruction and video processing	Not fertility-preserving
Prokopetc et al., 2015 [24]	Uterine laparoscopy	Computer vision	Technical study	95 images	Uterus and FU-junction detection	No clinical outcomes
François et al., 2020 [23]	Uterine laparoscopy	Computer vision	Technical/user study	3818 images	Automated contour detection for AR registration	No patient outcomes
Madad Zadeh et al., 2023 [25]	Gynecologic laparoscopy	Dataset/AI	Dataset study	3800 images	AR-enabling dataset	No clinical outcomes

Abbreviations: AI, artificial intelligence; AR, augmented reality; FU, fallopian tube-uterus; VR, virtual reality. Relationship to other tables: Table 4 is a subset of Table 2, restricted to endometriosis-related and enabling computer vision records; no record appears here that is not also in Table 2. Records are assigned to evidence streams in Table 2 and in Supplementary Table S3.

Bourdel et al. also reported AR visualization of adenomyomas in two cases, using T2-weighted MRI to generate models of the uterus, uterine cavity, and adenomyoma that were fused with laparoscopic video [6]. This is clinically important because adenomyomectomy requires awareness of both lesion position and the endometrial cavity. However, the evidence remains limited to a very small case-based report.

Chauvet et al. extended AR myomectomy by integrating diffusion tensor imaging and tractography to visualize uterine muscle fiber orientation during laparoscopic myomectomy [5]. This approach may support incision planning, but the proposed benefit for uterine scar quality remains hypothetical because scar healing, obstetric safety, and fertility outcomes were not evaluated.

Akladios et al. evaluated AR-assisted ureter localization in a gynecologic laparoscopic animal model, with surgeon video assessment suggesting improved ureter recognition compared with direct vision [18]. Although not a myomectomy or endometriosis clinical outcome study, it addresses a key technical problem in complex pelvic surgery: localization of hidden structures. Its applicability to fertility-preserving human surgery remains indirect.

The most clinically informative AR study was the retrospective matched case–control study by Comptour et al. [21]. This study included 17 AR-assisted laparoscopic myomectomy or adenomyomectomy cases and 17 controls. AR did not prolong operative time, and no intraoperative or postoperative complications were reported in either group. Nevertheless, the study was small, non-randomized, and not powered for reproductive outcomes. Direct and adjacent evidence in myomectomy and adenomyomectomy is summarized in Table 3.

### 3.4. Mixed Reality in Myomectomy

Of the two mixed-reality records, one involved an actual patient ([19]; Stream A) and one was an ex vivo model evaluation ([20]; Stream B). Two studies evaluated MR in myomectomy. Ochi et al. reported MR-assisted laparoscopic myomectomy in a 42-year-old nulligravid patient with multiple fibroids [19]. MRI-based 3D holograms were created using HoloeyesXR and displayed using HoloLens. The system supported fibroid localization, incision planning, and awareness of the endometrium. The postoperative course was uneventful, but the evidence was limited to a single case.

Torabinia et al. evaluated an MR headset during mock laparoscopic myomectomy using an ex vivo uterine fibroid model [20]. MRI segmentation was used to create a holographic rendering displayed through Microsoft HoloLens 2. The physician assessment suggested improved spatial visualization compared with two-dimensional monitor review, but this was an ex vivo feasibility evaluation without human clinical outcomes.

These studies support workflow plausibility and spatial visualization, but they do not establish clinical superiority, complication reduction, or reproductive benefit. They are presented separately from true intraoperative AR overlay in Table 1, Table 2 and Table 3.

### 3.5. Computer Vision and AI as AR-Enabling Technologies

All records in this subsection are Stream B enabling technologies, with one exception: François et al. [23] evaluated their contour-detection system during ten live gynecologic laparoscopies, which places that record in Stream A. None of the records in this subsection report a patient outcome. Several studies addressed computer vision methods relevant to future AR automation. Collins et al. developed an AR-guided laparoscopic system for uterine surgery that fused preoperative MR or CT data with monocular laparoscopic video [22]. This addressed markerless registration and real-time tracking of the mobile uterus, a key barrier to intraoperative AR.

François et al. developed an automated method for detecting uterine occluding contours to support augmented laparoscopy [23]. Their dataset included 3818 labeled laparoscopic uterus images, and a user study involving 10 gynecologic laparoscopies and five surgeons showed reduced surgeon interaction time without loss of registration accuracy.

Prokopetc et al. developed automatic detection of the uterus and fallopian tube-uterus junctions in laparoscopic images to support registration and fusion between preoperative imaging and laparoscopic images [24]. Sato et al. explored computer vision in 19 total laparoscopic hysterectomy videos, including image matching and panoramic reconstruction, although ureter detection was not satisfactory [17]. Madad Zadeh et al. introduced SurgAI3.8K, a labeled gynecologic laparoscopy dataset intended to support automatic AR surgical guidance [25].

In endometriosis, Netter et al. developed a YOLOv5-based model for automatic visual recognition of endometriosis lesion classes in laparoscopic videos from 112 patients [7]. Leibetseder et al. evaluated Faster R-CNN and Mask R-CNN for endometriosis detection and localization in laparoscopic gynecology videos [8]. These studies are relevant as AR-enabling lesion-recognition technologies but do not constitute real-time AR navigation and did not evaluate patient outcomes. The endometriosis and computer vision evidence is summarized in Table 4.

### 3.6. Three-Dimensional Modeling and Surgical Planning

All five records in this subsection are Stream A: in each, a model derived from a patient’s own imaging was used in planning a real scheduled procedure. 3D modeling was evaluated in both myomectomy and endometriosis surgery. Flaxman et al. evaluated MRI-derived patient-specific 3D-printed models in seven complex multifibroid uterus cases, including five myomectomies [26]. Surgeons reported that model review altered the planned dissection route in 8 of 15 surgeon responses and rated the models highly for preoperative planning and intraoperative reference. The denominator of 15 is at the level of the surgeon–case response rather than the patient: five surgeons participated (two staff surgeons and three clinical fellows), and one staff surgeon together with one or two fellows was present for each case, so 15 assessments were returned across the 7 cases. Two of those 7 cases were hysterectomies rather than myomectomies, so five of the seven procedures were uterus-preserving.

Li et al. reported a single-center randomized controlled trial of 3D printing model-assisted single-port laparoscopic multiple myomectomy [27]. In the per-protocol analysis, 110 patients were included, with 55 in each group. The 3D model group had shorter operative time, reduced surgeon workload, higher surgical plan adherence, and modestly reduced blood loss compared with conventional planning. Complication rates were not significantly different. Favorable trends were reported for residual and recurrent fibroids, but these did not reach statistical significance. Fertility-specific outcomes such as pregnancy and live birth were not reported.

For endometriosis, Borghese et al. created MRI-based patient-specific 3D virtual models in seven women scheduled for minimally invasive surgery for rectosigmoid endometriosis [10]. Surgeons reported high correlation with intraoperative findings in all cases. Martel et al. evaluated MRI-based 3D/VR modeling in colorectal endometriosis and reported perceived planning usefulness among expert surgeons [9]. Zhang et al. evaluated 3D modeling of deep endometriosis from pelvic MRI through a retrospective feasibility study, expert evaluation, national survey, and preliminary prospective cases [11].

Taken together, these studies suggest that 3D modeling is currently more mature as a preoperative planning tool than AR is as a real-time intraoperative guidance system. However, the effect of 3D modeling on fertility, obstetric outcomes, recurrence, adhesions, and long-term reproductive safety remains insufficiently studied. The design-appropriate appraisal of these planning studies, together with that of all other included records, is summarized in Table 5 and presented at study level, with the rationale for each judgment, in Supplementary Table S3.

### 3.7. Fertility and Reproductive Outcomes

A major finding of this review is the near absence of fertility-specific outcomes. Despite the fertility-preserving context of myomectomy, adenomyomectomy, and endometriosis surgery, most studies did not report pregnancy, live birth, miscarriage, uterine rupture, postoperative uterine integrity, adhesion formation, or obstetric safety.

No included study reported a statistically significant improvement in fertility, pregnancy, perioperative, or complication outcomes attributable to AR, MR, computer vision, or AI, and no adverse or negative findings were selectively omitted from the included reports; where comparative data were available, between-group differences were generally non-significant or exploratory.

Li et al. evaluated 1-year fibroid residual and recurrence outcomes, but fertility outcomes were not reported [27]. Comptour et al. provided the most clinically relevant AR comparative evidence, but reproductive outcomes were exploratory and insufficient for inference [21]. The reproductive data reported in that study should be stated in full, since they are the only such data in the included evidence. Over a median postoperative follow-up of 18 months (range 4–35.5), 11 of the 17 AR patients (64.7%) and 12 of the 17 matched controls (70.6%) expressed a desire for pregnancy; of these, 6 (35.3%) and 5 (29.4%) respectively conceived (*p* = 1.00). One myoma recurrence occurred among controls and none among AR cases (*p* = 1.00). Desire for pregnancy and number of pregnancies were prespecified items in that study’s data-collection plan, but pregnancy was not among its stated primary or secondary endpoints, and the study was designed and powered to test non-inferiority of operative time. A six-versus-five comparison in a retrospective sample of 34 patients cannot support inference in either direction, and no live birth, miscarriage, uterine rupture, uterine-integrity, or obstetric outcome was reported. At present, AR, MR, computer vision, and 3D modeling remain fertility-relevant in concept but fertility-unproven in evidence. Key potential contributions and appropriate interpretations are summarized in Table 6, while the main evidence gaps are summarized in Table 7.

### 3.8. Quality and Applicability Appraisal

The structured appraisal confirmed that the evidence remains early in maturity. The RCT by Li et al. provided the strongest comparative evidence but was limited by single-center design, lack of blinding, retrospective registration, and absence of fertility endpoints [27]. The retrospective matched case–control AR study by Comptour et al. was clinically relevant but small and non-randomized [21]. Most AR and MR studies were case reports, experimental studies, or technical reports. AI and computer vision studies provided important enabling methods but generally lacked clinical outcome validation. The quality and applicability appraisal is summarized in Table 5.

Because the included studies spanned heterogeneous designs (one randomized controlled trial, one non-randomized comparative study, case reports and case series, and technical, dataset, and feasibility studies), a single per-study risk-of-bias instrument was not applicable across all records. Studies were therefore appraised in design-based groups, applying the most appropriate framework to each design (randomized-trial risk-of-bias domains, ROBINS-I, JBI critical appraisal principles, and structured feasibility criteria), as summarized in Table 5. In response to the review, the appraisal has been extended in two ways. First, a study-level appraisal is now presented for every one of the 20 included records in Supplementary Table S3, Panel A, stating the instrument applied, the judgment reached in each domain, and the basis for that judgement. Second, because the four instruments used are not directly comparable across designs, a cross-design grid of six domains that can be evaluated for every design—selection and representativeness, comparator or reference standard, directness of the evidence to the review question, objectivity of outcome measurement, completeness of outcome reporting and follow-up, and reporting of fertility-relevant outcomes—is presented as a color-coded grid in Supplementary Table S3, Panel B. That grid is intended as a visual summary of applicability and of the principal bias concerns; it does not replace the design-specific instruments, and it should be read together with Panel A. Two patterns are visible across the grid. Objectivity of outcome measurement is the most frequently compromised domain, because the dominant outcome in this literature is the operating surgeon’s own perception of usefulness, recorded without blinding. Reporting of fertility-relevant outcomes is absent in every record without exception, including the randomized trial.

### 3.9. Implementation Characteristics and Completeness of Technical Reporting

A further consequence of the immaturity of this literature is that the information a center would need in order to reproduce or adopt any of these workflows is frequently not reported. Supplementary Table S5 charts, for each included record, the software or segmentation platform used, the display or headset hardware, the source imaging modality, and the registration or alignment method. The pattern of what is and is not reported is itself a finding rather than a limitation of the charting. Source imaging modality is reported almost universally, and the registration or alignment approach is described in principle by most records that have one. Two gaps are systematic. First, and most consequentially, a quantitative accuracy metric is reported by only a small minority of records, so for most of this literature the reader is told how registration was attempted but not how well it worked. Second, the display or headset hardware and the specific software platform and version are frequently unnamed, which prevents a reader from establishing what was actually used. Where a field could not be established from the published report it is recorded as not reported (NR). This has a direct practical consequence. A described registration method without a measured accuracy cannot be assessed for fitness in fertility-preserving surgery, where the tolerance for misregistration is set by the proximity of the endometrial cavity and the ureter rather than by what is technically convenient; and an unnamed platform cannot be procured or reproduced. For most of the workflows in this review, including several that have been applied in patients, published reporting is therefore insufficient to permit independent technical replication or informed adoption.

## 4. Discussion

This scoping review shows that AR, MR, computer vision, and 3D modeling are promising but still investigational in fertility-preserving minimally invasive gynecologic surgery. The field has moved beyond purely conceptual work, but most evidence remains technical, feasibility-based, or early clinical.

A key methodological issue is terminology. The distinction between these technologies is set out in Section 1 and in Table 1 and is not restated here. Treating them as equivalent can overstate the maturity of the field. For this reason, the present review classified studies by technology type and separated direct AR evidence from enabling computer vision and 3D modeling studies. This classification is shown in Table 1, and the complete evidence map is presented in Table 2.

The available evidence is strongest in myomectomy and adenomyomectomy, where the problem of hidden or poorly demarcated uterine pathology is well suited to image guidance. MRI-based models may help the surgeon understand the location of intramural myomas, adenomyomas, and the endometrial cavity [3,4,5,6,21]. This is clinically meaningful because fertility-preserving uterine surgery depends on selective excision, careful reconstruction, and preservation of functional uterine tissue.

However, feasibility is not the same as effectiveness. Most AR and MR studies demonstrate that these technologies can be used, but few show that they improve outcomes. The field currently has limited comparative AR evidence, no randomized trial of real-time AR overlay, and minimal fertility follow-up. Therefore, statements about reduced complications, reduced tissue trauma, improved scar quality, lower cavity-entry risk, or improved fertility should be framed as hypotheses rather than established effects.

The strongest comparative evidence in this review concerns 3D modeling rather than real-time AR [27]. The operative-process findings of that trial are reported in Section 3.6 and are not repeated here. This is clinically relevant, but it also highlights an important point: preoperative 3D planning may currently be more mature than intraoperative AR in reproductive gynecologic surgery. Even in this RCT, fertility outcomes were not reported.

Endometriosis surgery represents a different challenge. In deep endometriosis, the problem is often not localization of a single hidden lesion but reconstruction of distorted pelvic anatomy. Three-dimensional modeling may support understanding of lesion extent and relationships to bowel, ureter, bladder, and pelvic sidewall structures [9,10,11]. AI lesion recognition may eventually contribute to intraoperative awareness [7,8]. At present, however, validated real-time AR navigation for endometriosis surgery has not yet been established.

A further limitation is pelvic tissue deformation. Most AR systems rely on preoperative imaging, but the uterus and surrounding pelvic structures change shape during manipulation, incision, traction, dissection, suturing, and reconstruction. Endometriosis surgery adds another layer of complexity because adhesiolysis and mobilization progressively change the operative field. Static registration may therefore become less accurate as the operation progresses.

This issue is especially important in fertility-preserving surgery because small inaccuracies may have consequences beyond the immediate procedure. A suboptimal uterine incision, excessive myometrial resection, avoidable cavity entry, ureteral injury, bowel injury, or poor reconstruction may affect recovery, future fertility, pregnancy safety, or obstetric outcomes. For AR to become clinically useful, it must be accurate, stable, interpretable, and integrated into the surgical workflow without increasing cognitive burden.

The absence of fertility-specific outcomes is the most important limitation of the field. If these technologies are proposed as fertility-preserving tools, they must be evaluated using fertility-relevant endpoints. Technical feasibility alone is not sufficient. Future studies should report uterine cavity entry, quality of uterine reconstruction, residual disease, recurrence, adhesions, pregnancy, live birth, miscarriage, uterine rupture, and obstetric outcomes. To state the finding without qualification, because it is arguably the single most important result of this review: there is at present no evidence that AR, MR, computer vision, AI, or 3D modeling improves fertility, live birth rate, pregnancy rate, miscarriage rate, uterine rupture risk, obstetric outcome, or any measure of long-term reproductive safety in this surgical population. This is not a matter of inconsistent or conflicting findings. Reproductive outcomes are almost never reported. Exactly one of the 20 included records reports any reproductive outcome: over a median follow-up of 18 months, the matched AR series reports that 6 of the 11 AR patients who expressed a desire for pregnancy conceived, compared with 5 of the 12 such patients among matched controls (*p* = 1.00), with one myoma recurrence in the control group and none in the AR group (*p* = 1.00) [21]. Those data are the entire reproductive evidence base of this field. They derive from a retrospective sample of 34 patients designed and powered to test non-inferiority of operative time rather than any reproductive endpoint, pregnancy was not among the study’s stated primary or secondary endpoints, and the confidence around a six-versus-five comparison is far too wide to support inference in either direction. No included record—including the only randomized controlled trial in the review, which reports no reproductive outcome of any kind—reports live birth, miscarriage, uterine rupture, postoperative uterine integrity, or any obstetric or long-term reproductive-safety outcome (Supplementary Table S3, Panel B). Descriptions of these technologies as “promising” should therefore be read strictly as referring to technical feasibility, to the operative process, and to surgeon-reported planning utility, and not as referring to reproductive benefit of any kind. The scope of the term “fertility-preserving”. This creates a discrepancy between the title and objectives of this review and the evidence it was able to map, and the discrepancy should be stated openly rather than left implicit. The review deliberately targeted fertility-preserving surgery, yet the overwhelming majority of the included studies report no fertility-related endpoint. In this review, therefore, “fertility-preserving” denotes the clinical context in which the surgery is performed—uterus-preserving, anatomy-preserving procedures in women of reproductive age for whom future fertility is a treatment goal—and not a demonstrated reproductive benefit of the technologies under study. The term describes the population and the surgical intent, not the effect. Recognizing this explicitly is useful in two ways: it prevents the reader from over-reading the included evidence, and it identifies precisely where the field must go next, since a technology promoted on fertility-preservation grounds should ultimately be judged on fertility-relevant endpoints rather than on operative-process surrogates. Cost, accessibility, learning curve, and implementation barriers. Alongside the evidential questions, four practical barriers determine whether any of these technologies can reach routine clinical use, and none of them is adequately addressed in the literature mapped here. Cost and reimbursement: no included study reports a formal economic evaluation, a cost-effectiveness analysis, or a per-case cost. The cost structures differ substantially between the technology classes—patient-specific 3D printing incurs a recurring per-case materials, segmentation-labor, and turnaround cost, whereas AR and MR systems incur a large capital and software-licensing cost that is then amortized across cases—and these two profiles have different implications for adoption in publicly funded systems. Accessibility and infrastructure: every workflow in this review requires high-quality cross-sectional imaging, expert segmentation, and, for AR and MR, integration with the existing laparoscopic stack. Segmentation in particular has generally been performed by engineering or radiology collaborators rather than by the surgical team, which concentrates these workflows in centers with an embedded technical partnership and limits transferability. Learning curve: no included study reports a formal learning-curve analysis, and the perceived-usefulness outcomes that dominate this literature were generally reported by the developers or by expert users at developing centers, so they are unlikely to represent the experience of a surgeon adopting the technology for the first time. Workflow burden: additional preoperative segmentation time, intraoperative set-up time, and the cognitive load of attending to an overlay while operating are inconsistently reported; where objectively measured, the relevant findings concerned surgeon interaction time and workload rather than patient outcome [23,27]. The reporting gaps underlying these four barriers are documented record by record in Supplementary Table S5. Until they are addressed, the principal obstacle to clinical translation in this field is likely to be implementation rather than technical capability.

### Limitations of This Review

Several methodological limitations of the review process should be acknowledged. The search was restricted to English-language records and to three sources (PubMed/MEDLINE, Scopus, and Google Scholar), with Google Scholar used only as a supplemental source and a fixed number of results screened per query; relevant gray literature or non-indexed reports may therefore have been missed. Study selection and data charting were performed by the first author and verified by a senior author rather than by two fully independent reviewers, and the review was not registered and had no published a priori protocol. Because of the heterogeneity of study designs, technologies, procedures, and outcomes, no quantitative synthesis or formal certainty-of-evidence grading was undertaken, and the synthesis is necessarily narrative and descriptive. Finally, most included records were small, early-phase, feasibility, or technical studies, so the findings reflect the current immaturity of the evidence base rather than established effectiveness. Several of these limitations warrant fuller statement. Database coverage and publication type. The search was confined to PubMed/MEDLINE, Scopus, and Google Scholar, and conference papers were excluded a priori. Both decisions systematically under-represent one part of this literature. A substantial proportion of the primary technical work on surgical AR, intraoperative registration, and computer vision is first published in peer-reviewed conference proceedings—notably IPCAI, MICCAI, and IEEE ISMAR—which are indexed in IEEE Xplore and the ACM Digital Library rather than in the biomedical databases searched here; Embase, Web of Science, and the Cochrane Library were likewise not searched. The eligibility criteria were framed around clinically oriented evidence in benign fertility-preserving gynecologic surgery, and the peer-reviewed journal literature indexed in PubMed/MEDLINE and Scopus was judged to capture that evidence; nevertheless, the consequence is a genuine under-mapping of the engineering and computer-science layer of this field. The enabling-technology component of this review should accordingly be read as an incomplete map rather than an exhaustive one, and the absence of an identified study should not be taken as evidence that the underlying technical capability does not exist. A future review of the technical literature, searching IEEE Xplore, the ACM Digital Library, Embase, and Web of Science and including full conference papers, would complement the present clinically oriented synthesis. Reproducibility of supplementary searching. Google Scholar results were screened by relevance ranking to a fixed depth of 25 records per query, which is not exactly reproducible. As reported in Section 2.3, this source contributed no unique included record, so the reproducible PubMed/MEDLINE and Scopus searches account for the entire included evidence base. Language restriction. Eligibility was restricted to English-language records. Language bias cannot be excluded, and relevant work published in other languages—plausibly including Chinese, Japanese, French, and Italian reports, given the geographical distribution of the included studies—may have been missed. Screening and charting procedure. As set out in Section 2.6, screening and charting were performed by a single reviewer with subsequent senior verification rather than independently and in duplicate, and no inter-reviewer agreement statistic is reported. Single-screener designs carry a recognized risk of records being missed at the title-and-abstract stage. Absence of a registered protocol and of a record-level exclusion log. The review was not prospectively registered, and although the methods were specified a priori (Section 2.1) and the eligibility parameters, search strategies, and exclusion criteria are reported in full in Section 2.1 to Section 2.6 and in Supplementary Tables S2 and S4, the absence of a public protocol means that adherence to them cannot be verified externally. A second and distinct limitation concerns the audit trail for study selection. Reasons for exclusion at the full-text stage were documented prospectively at the level of exclusion category, and a record-level bibliographic log of the 56 excluded full texts was not retained. Supplementary Table S2 therefore reports the operational criterion and the count for each exclusion category, but does not identify the excluded records individually, and aggregate categories and counts of this kind do not constitute an audit trail equivalent to a record-level list. The practical consequence should be stated plainly: a reader can apply the stated criteria to the same retrieved set and arrive at the same 20 included records, but cannot re-audit the 56 individual exclusion decisions that were actually made. Because the log was not retained at the time, this limitation cannot now be resolved, and any list assembled retrospectively would be a reconstruction rather than a record of the original screening. It is disclosed here without qualification. Prospective registration and retention of a record-level screening log have been adopted for subsequent reviews by this group.

## 5. Future Directions

Future research should move from feasibility reporting toward clinically meaningful evaluation. Priority outcomes should include registration accuracy, operative time, blood loss, complications, conversion, cavity entry, completeness of lesion removal, surgeon workload, workflow burden, cost, and fertility-related outcomes. Given that fertility-related outcomes are absent from the entire present evidence base, they should be treated as the primary rather than a secondary endpoint in studies of technologies advanced on fertility-preservation grounds. Reporting should also be complete enough to permit technical replication: the software platform and version, the display hardware, the source imaging modality, the registration or alignment method, and a quantitative registration-accuracy metric should be stated in every report, since these items are frequently missing at present (Supplementary Table S5).

Dynamic registration remains a key technical priority. The uterus changes shape with manipulation, traction, incision, myoma enucleation, and suturing. Endometriosis surgery adds further complexity because adhesiolysis and mobilization progressively change anatomy. Future AR systems will need to account for deformable anatomy in real time.

AI and computer vision are likely to play a central role in future AR systems. Automated segmentation, organ recognition, lesion detection, and tracking may reduce manual input and improve workflow integration. However, these systems must be validated clinically before they can guide surgical decisions.

Future studies should also clearly separate technology types. True intraoperative AR overlay, MR visualization, 3D surgical planning, and AI lesion recognition should be reported as distinct interventions. This will improve reproducibility and prevent overstatement of evidence.

Most importantly, future studies should be fertility-centered. If AR, MR, AI, or 3D modeling is promoted as fertility-preserving, its value should be measured in outcomes that matter to reproductive patients.

## 6. Conclusions

AR, MR, computer vision, and 3D modeling are investigational adjuncts in fertility-preserving minimally invasive gynecologic surgery, at an early stage of clinical evaluation. Of the 20 records identified, eleven involved application in patients, of which only two were comparative and only one reported any reproductive outcome (Section 3.2). Current evidence is strongest for technical feasibility and preoperative 3D planning, especially in myomectomy and deep endometriosis. Direct intraoperative AR evidence remains early and is supported mainly by technical studies, case reports, and one small retrospective comparative study.

The available literature does not yet prove that AR or related technologies improve fertility, reduce complications, improve uterine healing, or improve obstetric outcomes. The position should be stated precisely. For live birth, miscarriage, uterine rupture, postoperative uterine integrity, obstetric outcome, and long-term reproductive safety there is no evidence at all, because no included study reported these outcomes. For pregnancy, a single retrospective matched series reports rates that did not differ between groups and that were not a stated endpoint of that study [21]. On either reading, no included study demonstrates a reproductive benefit. This absence of evidence, rather than any positive finding, is the principal result of this review, and the term “fertility-preserving” in the title of this review refers to the clinical context of the surgery and not to a demonstrated reproductive benefit. Proposed benefits such as improved lesion localization, reduced blind dissection, better cavity awareness, or safer endometriosis surgery should be framed as plausible hypotheses rather than established effects.

Future research should use prospective controlled designs, standardized reporting, validated technical metrics, and fertility-relevant endpoints. Until such data are available, these technologies should be considered investigational tools of unproven clinical benefit rather than established standards of care in fertility-preserving gynecologic surgery. Adoption decisions should additionally take account of cost, infrastructure, learning curve, and workflow burden, none of which is adequately characterized in the present literature (Section 4).

## Figures and Tables

**Table 1:** Operational Categories of Included Technologies.

Category	Definition	Examples in This Review	Interpretation
True intraoperative AR overlay	Real-time fusion of virtual imaging data with laparoscopic video	MRI-derived uterus/myoma/adenomyoma models fused with laparoscopy	Most direct AR evidence, but clinical outcomes remain limited
Mixed reality visualization	Display of holographic or virtual models within the operative environment	HoloLens/HoloeyesXR in myomectomy	Useful for spatial understanding, but not equivalent to registered AR overlay
AI and computer vision enabling technologies	Automated recognition, segmentation, tracking, contour detection, or lesion detection	Uterus contour detection, endometriosis lesion recognition, SurgAI3.8K	Foundational for future AR systems, but not direct clinical AR evidence
Preoperative 3D modeling/3D printing/VR planning	Creation of 3D models or virtual models for planning or surgical rehearsal	3D-printed fibroid models, VR models for deep endometriosis	More mature for surgical planning than real-time intraoperative AR

Abbreviations: AI, artificial intelligence; AR, augmented reality; VR, virtual reality.

**Table 2:** Evidence Map of the 20 Included Records.

No.	Study	Technology Category	Procedure/Context	Design	Sample	Main Contribution	Main Limitation	Evidence Level
1	Bourdel et al., 2017 [3]	AR overlay—Stream B	Myomectomy model	Experimental user study	10 residents	AR improved myoma localization accuracy in uterine model	No patient outcomes	V
2	Bourdel et al., 2017 [4]	AR overlay—Stream A	Laparoscopic myomectomy	Clinical feasibility report	3 patients	Real-time MRI-based myoma localization	Very small sample	V
3	Bourdel et al., 2019 [6]	AR overlay—Stream A	Adenomyomectomy	Case report	2 patients	AR localization of adenomyoma and uterine cavity	No comparator	V
4	Chauvet et al., 2020 [5]	AR overlay with DTI—Stream A	Laparoscopic myomectomy	Case reports	2 patients	Visualization of uterine fiber orientation	No clinical validation	V
5	Akladios et al., 2020 [18]	AR overlay—Stream B	Ureter localization in gynecologic laparoscopy	Animal model/surgeon evaluation	5 pigs; 58 surgeons	AR improved ureter recognition in video assessment	Indirect animal-model evidence	V
6	Ochi et al., 2023 [19]	MR visualization—Stream A	Laparoscopic myomectomy	Case report	1 patient	MRI-based holographic planning and incision guidance	Single case	V
7	Torabinia et al., 2022 [20]	MR visualization—Stream B	Mock laparoscopic myomectomy	Ex vivo model	1 model	HoloLens 2 improved perceived spatial visualization	No human outcomes	V
8	Comptour et al., 2025 [21]	AR overlay—Stream A	Myomectomy/adenomyomectomy	Retrospective matched case–control	34 patients	AR did not prolong operative time and no added adverse events were reported	Small, non-randomized	III
9	Collins et al., 2021 [22]	AR registration/tracking—Stream B	Uterine laparoscopy	Technical development	NR	Markerless AR registration of mobile uterus	Technical evidence only	V
10	François et al., 2020 [23]	AI and computer vision—Stream A	Uterus contour detection	Technical study with user study	3818 images; 5 surgeons	Automated occluding-contour detection reduced surgeon interaction time	No clinical outcomes	V
11	Prokopetc et al., 2015 [24]	AI and computer vision—Stream B	Uterus and FU-junction detection	Technical study	95 images	Automated uterus and FU-junction detection for registration	Technical dataset only	V
12	Sato et al., 2019 [17]	Computer vision—Stream B	Total laparoscopic hysterectomy	Preliminary video study	19 cases	Explored OpenCV-based gynecologic laparoscopy analysis	Not fertility-preserving	V
13	Madad Zadeh et al., 2023 [25]	AI and computer vision dataset—Stream B	Gynecologic laparoscopy	Dataset study	3800 images	Dataset for automatic AR surgical guidance	No patient outcomes	V
14	Netter et al., 2025 [7]	AI lesion recognition—Stream B	Endometriosis laparoscopy	Multicenter proof-of-concept	112 videos	YOLOv5 recognition of endometriosis lesion classes	Variable performance	V
15	Leibetseder et al., 2022 [8]	AI lesion localization—Stream B	Endometriosis laparoscopy	Technical AI study	Video dataset	Faster R-CNN/Mask R-CNN lesion localization	Moderate performance	V
16	Flaxman et al., 2024 [26]	3D printing—Stream A	Multifibroid uterus	Surgical planning evaluation	7 cases	3D models changed planning and dissection route	No comparative outcomes	IV
17	Li et al., 2026 [27]	3D printing—Stream A	Single-port laparoscopic multiple myomectomy	Randomized controlled trial	133 randomized and analyzed (intention to treat); 110 per protocol (55/group)	Reduced operative time and surgeon workload	Single-center; no fertility outcomes	II
18	Martel et al., 2026 [9]	3D/VR modeling—Stream A	Colorectal endometriosis	Retrospective feasibility/expert evaluation	14 models	Supported surgical planning perception	No outcome comparison	IV
19	Borghese et al., 2022 [10]	3D virtual modeling—Stream A	Rectosigmoid endometriosis	Prospective pilot cohort	7 women	High correlation with intraoperative findings	Small subjective study	IV
20	Zhang et al., 2026 [11]	3D modeling—Stream A	Deep endometriosis	Feasibility/survey/preliminary prospective	NR	MRI-based 3D modeling of deep endometriosis	No real-time AR or fertility outcomes	IV

Abbreviations: AR, augmented reality; DTI, diffusion tensor imaging; FU, fallopian tube-uterus; MR, mixed reality; NR, not reported; VR, virtual reality. Evidence stream (defined in Section 3.2): Stream A, the technology was applied in the care of actual patients, either intraoperatively or in the preoperative planning of a real scheduled procedure (n = 11); Stream B, the technology was developed or validated on image datasets, retrospective operative video, bench or ex vivo models, or animal models, without deployment in patient care (n = 9). Evidence levels (defined in full in Section 2.10): II, individual randomized controlled trial; III, non-randomized comparative study with a concurrent or matched comparator; IV, single-arm cohort, case series, feasibility study in patients, or structured surgeon-assessment study without a comparator; V, case report, technical development report, dataset or annotation study, bench or ex vivo study, animal-model study, or mechanism-based reasoning. Level I (systematic review or meta-analysis of randomized trials) was not met by any included record. Levels describe study design and directness of evidence to the review question; they are not certainty-of-evidence grades and were not used to weight or exclude records.

**Table 5:** Structured Quality and Applicability Appraisal.

Study Group	Appraisal Approach	Strengths	Main Concerns	Interpretation
Li et al., 2026 [27]	RCT risk-of-bias domains	Randomized design; comparative outcomes; follow-up	Single-center; unblinded; retrospective registration; fertility outcomes absent	Strongest comparative evidence, but not AR-specific
Comptour et al., 2025 [21]	ROBINS-I domains	Matched comparative AR study; direct uterine surgery relevance	Small sample; non-randomized; limited reproductive follow-up	Most clinically informative AR study
Bourdel et al., 2017/2019, Chauvet et al., 2020, Ochi et al., 2023 [4,5,6,19]	JBI case report principles	Direct clinical relevance	Very small samples; no comparators	Feasibility evidence only
Bourdel et al., 2017, Akladios et al., 2020, Torabinia et al., 2022 [3,18,20]	Technical feasibility appraisal	Addresses key technical issues	Animal/ex vivo setting; no patient outcomes	Indirect but useful
Collins, Francois, Prokopetc, Sato, Madad Zadeh, Netter, Leibetseder [7,8,17,22,23,24,25]	Technical/AI feasibility appraisal	Important enabling technology	No fertility outcomes; limited clinical validation	Foundational evidence
Flaxman, Martel, Borghese, Zhang [9,10,11,26]	Feasibility/planning appraisal	Relevant planning data	Mostly subjective or small cohorts	Planning value plausible but outcome effect unproven

Appraisal instruments: RCT, randomized-trial risk-of-bias domains; ROBINS-I, Risk Of Bias In Non-randomized Studies of Interventions; JBI, Joanna Briggs Institute critical appraisal principles. Studies are appraised in design-based groups here; the study-level appraisal for each of the 20 included records, the cross-design domain grid, and the record-to-table correspondence are provided in Supplementary Table S3 (Panels A–C). Evidence levels are defined in full in Section 2.10 and in the footnotes to Table 2 and Table 3.

**Table 6:** Potential Contributions and Correct Interpretation.

Surgical Challenge	Technology	Potential Contribution	Correct Interpretation
Occult intramural myoma localization	AR/MR/3D modeling	Improved spatial awareness and incision planning	Feasible, but clinical superiority not proven
Adenomyoma boundary and cavity awareness	AR/3D modeling	Better understanding of lesion-cavity relationship	Strong rationale, limited data
Multiple fibroid removal sequence	3D printing	Preoperative rehearsal and removal sequence planning	Supported by one RCT, fertility outcomes absent
Mobile uterus registration	AR/computer vision	Markerless tracking and model fusion	Technical feasibility shown
Endometriosis lesion recognition	AI and computer vision	Automated lesion detection/localization	Early technical evidence
Distorted deep endometriosis anatomy	3D/VR modeling	Surgical planning and anatomical communication	Planning value plausible
Surgeon workload	3D printing/AR automation	Reduced planning or interaction burden	Evidence strongest for 3D myomectomy RCT
Fertility preservation	All technologies	Potential tissue-sparing relevance	Not proven by current evidence

**Table 7:** Key Evidence Gaps.

Domain	Current State	Why It Matters	Future Research Need
Fertility outcomes	Rarely reported	Central to review question	Pregnancy, live birth, miscarriage, uterine rupture
Registration accuracy	Inconsistently reported	Misregistration may mislead	Standardized AR accuracy metrics
Deformable anatomy	Limited solutions	Uterus changes during surgery	Dynamic registration
Comparative AR evidence	Sparse	Cannot infer superiority	Prospective controlled studies
Long-term outcomes	Limited	Recurrence and obstetric safety matter	Longitudinal follow-up
Workflow burden	Incompletely assessed	Adoption depends on usability	Human-factors studies
Cost-effectiveness	Not established	3D printing/AR may require resources	Economic analyses
Standardization	Terminology varies	Comparability is poor	Consensus reporting framework

## Data Availability

No new data were created or analyzed in this study. Data sharing is not applicable to this article.

## Supplementary Materials

The following supporting information can be downloaded at: https://www.mdpi.com/article/10.3390/medicina62091790/s1, Table S1, completed PRISMA-ScR (PRISMA Extension for Scoping Reviews) checklist; Table S2, full-text exclusions with the operational criterion and count for each exclusion category; Table S3, study-level critical appraisal (Panel A, design-specific appraisal; Panel B, cross-design domain grid; Panel C, correspondence between the included records and Table 2, Table 3 and Table 4); Table S4, complete search strategies as executed in each source; Table S5, implementation characteristics of the included studies.

## Abbreviations

AI, artificial intelligence; AR, augmented reality; CT, computed tomography; DTI, diffusion tensor imaging; FU, fallopian tube–uterus; ICG, indocyanine green; JBI, Joanna Briggs Institute; MR, mixed reality; MRI, magnetic resonance imaging; NA, not applicable; NR, not reported; OCEBM, Oxford Centre for Evidence-Based Medicine; PRISMA, Preferred Reporting Items for Systematic Reviews and Meta-Analyses; PRISMA-ScR, PRISMA Extension for Scoping Reviews; RCT, randomized controlled trial; R-CNN, region-based convolutional neural network; ROBINS-I, Risk Of Bias In Non-randomized Studies of Interventions; VR, virtual reality; YOLO, You Only Look Once; 3D, three-dimensional.
