# Supplementary material for: Augmented Reality, Mixed Reality, Computer Vision, and Three-Dimensional Modeling in Fertility-Preserving Minimally Invasive Gynecologic Surgery: A Scoping Review with Narrative Synthesis
Source: Medicina (Kaunas). 2026 Sep 17;62(9):1790. doi: 10.3390/medicina62091790 (PMC13609672; doi:10.3390/medicina62091790)
Supplement: Supplementary file 1 [file medicina-62-01790-s001.zip › Table_S1_PRISMA-ScR_FINAL.pdf]

# Supplementary Material – Table S1

## PRISMA-ScR (PRISMA Extension for Scoping Reviews) Checklist

*Manuscript: "Augmented Reality, Mixed Reality, Computer Vision, and Three-Dimensional Modeling in Fertility-Preserving Minimally Invasive Gynecologic Surgery: A Scoping Review with Narrative Synthesis" (medicina-4409602).*

Checklist source: Tricco AC, Lillie E, Zarin W, et al. PRISMA Extension for Scoping Reviews (PRISMA-ScR): Checklist and Explanation. Ann Intern Med. 2018;169(7):467–473. Section references correspond to the second revision of the manuscript.

| Section | Item | PRISMA-ScR checklist item | Reported | Location in the revised manuscript |
|---|---|---|---|---|
| **TITLE** | 1 | Identify the report as a scoping review. | Yes | Title page ("…: A Scoping Review with Narrative Synthesis"). |
| **ABSTRACT** | 2 | Provide a structured summary including, as applicable, background, objectives, eligibility criteria, sources of evidence, charting methods, results, and conclusions. | Yes | Abstract (rewritten and shortened in this revision; Background and Objectives; Materials and Methods; Results; Conclusions). |
| **INTRODUCTION** | 3 | Describe the rationale for the review in the context of what is already known. | Yes | Section 1, Introduction (expanded in this revision with the surgeon-facing rationale and its evidential limits). |
| | 4 | Provide an explicit statement of the questions and objectives addressed, with reference to key elements such as population, concept, and context. | Yes | Section 1, final paragraph; population–concept–context framework in Section 2.2. |
| **METHODS** | 5 | Indicate whether a review protocol exists; state if and where it can be accessed; provide registration information if available. | Yes | Section 2.1: not registered and no protocol published; the reason for non-registration, and the a priori specification of methods, are now stated in full. |
| | 6 | Specify characteristics of the sources of evidence used as eligibility criteria, and provide a rationale. | Yes | Sections 2.2, 2.4, 2.5; language and publication-status limits in Section 2.3; rationale and consequences in Section 4 (Limitations). |
| | 7 | Describe all information sources searched, with dates of coverage, and the date the most recent search was executed. | Yes | Section 2.3 (PubMed/MEDLINE, Scopus, Google Scholar; inception to the search date; primary search 11 February 2026, confirmatory re-run 16 May 2026). |
| | 8 | Present the full electronic search strategy for at least one database, including any limits used, such that it could be repeated. | Yes | Section 2.3 (concise description of the sources, search period, and search approach); the complete strings as executed, with the rationale for the syntax differences between platforms, are reproduced in Supplementary Table S4. |
| | 9 | State the process for selecting sources of evidence. | Yes | Section 2.6, Study Selection (now states that screening was single-reviewer with senior verification, not independent and in duplicate, and that no agreement statistic is reported). |
| | 10 | Describe the methods of charting data and any processes for obtaining and confirming data from investigators. | Yes | Sections 2.7 (Data Extraction) and 2.8 (Technology Classification); Table 1 relocated to follow Section 2.8. |

| Section | Item | PRISMA-ScR checklist item | Reported | Location in the revised manuscript |
|---|---|---|---|---|
| | 11 | List and define all variables for which data were sought, and any assumptions and simplifications made. | Yes | Section 2.7 (variables charted; NR/NA conventions); implementation variables charted in Supplementary Table S5. |
| | 12 | If done, provide a rationale for a critical appraisal of included sources; describe the methods used and how the information was used in synthesis. | Yes | Sections 2.9 (Quality and Applicability Appraisal) and 2.10 (Evidence Level Classification, now with the full Level I–V hierarchy defined). |
| | 13 | Describe the methods of handling and summarizing the charted data. | Yes | Section 2.8; narrative-synthesis approach; Section 3.1 (no meta-analysis owing to heterogeneity); evidence-stream framework in Section 3.2. |
| **RESULTS** | 14 | Give numbers of sources screened, assessed for eligibility, and included, with reasons for exclusion at each stage, ideally using a flow diagram. | Yes | Section 3.1 and Figure 1 (flow diagram); reasons for the 56 full-text exclusions now set out with the operational criterion and count for each category in Supplementary Table S2. |
| | 15 | For each source of evidence, present characteristics for which data were charted, and provide citations. | Yes | Section 3.2; Tables 2, 3, and 4; record-to-table correspondence in Supplementary Table S3, Panel C. |
| | 16 | If done, present data on the critical appraisal of included sources. | Yes | Section 3.8; Table 5 (now containing all 20 records); study-level appraisal in Supplementary Table S3, Panels A and B. |
| | 17 | For each included source, present the relevant charted data relating to the review questions and objectives. | Yes | Sections 3.3–3.7; Tables 2–4 and 6; implementation characteristics in Supplementary Table S5 (Section 3.9). |
| | 18 | Summarize and present the charting results as they relate to the review questions and objectives. | Yes | Sections 3.2–3.9; Tables 6 and 7. |
| **DISCUSSION** | 19 | Summarize the main results, link them to the review questions and objectives, and consider relevance to key groups. | Yes | Section 4, Discussion (opening paragraphs); new subsections on the scope of the term "fertility-preserving" and on cost, accessibility, learning curve, and implementation barriers. |
| | 20 | Discuss the limitations of the scoping review process. | Yes | Section 4, "Limitations of this review", substantially expanded in this revision (database coverage, conference-paper exclusion, supplementary-search reproducibility, language restriction, screening procedure, non-registration, and record-level exclusion log). |
| | 21 | Provide a general interpretation of the results with respect to the review questions and objectives, and potential implications or next steps. | Yes | Section 5 (Future Directions) and Section 6 (Conclusions), both moderated in this revision to state the absence of fertility-related evidence explicitly. |

| Section | Item | PRISMA-ScR checklist item | Reported | Location in the revised manuscript |
|---|---|---|---|---|
| **FUNDING** | 22 | Describe sources of funding for the included sources of evidence and for the scoping review, and the role of the funders. | Yes | Funding statement ("This research received no external funding"). |

Note. Items 12 and 16 (critical appraisal of sources of evidence) are optional in PRISMA-ScR. They were completed because a structured quality and applicability appraisal was performed (Sections 2.9, 2.10, and 3.8; Table 5; Supplementary Table S3). Item 14 requires reasons for exclusion to be reported at each stage at which eligibility is formally assessed; these are reported at exclusion-category level in Supplementary Table S2 and Figure 1, and the absence of a record-level exclusion log is declared as a limitation in Section 4.
