# Supplementary material for: Augmented Reality, Mixed Reality, Computer Vision, and Three-Dimensional Modeling in Fertility-Preserving Minimally Invasive Gynecologic Surgery: A Scoping Review with Narrative Synthesis
Source: Medicina (Kaunas). 2026 Sep 17;62(9):1790. doi: 10.3390/medicina62091790 (PMC13609672; doi:10.3390/medicina62091790)
Supplement: Supplementary file 1 [file medicina-62-01790-s001.zip › Table_S2_Excluded_Studies_FINAL.pdf]

Supplementary Material – Table S2

Full-Text Exclusions: Operational Criteria and Counts

Manuscript: "Augmented Reality, Mixed Reality, Computer Vision, and Three-Dimensional Modeling in Fertility-Preserving Minimally Invasive Gynecologic Surgery: A Scoping Review with Narrative Synthesis" (medicina-4409602).

Panel A. Reasons for exclusion at full-text assessment (n = 56)

Each category below corresponds to one line of the exclusion box in the PRISMA flow diagram (Figure 1). For each category, the operational criterion that was applied when a full text was assessed is stated in full, so that a reader applying the same criterion to the same retrieved set would reach the same decision.

Exclusion category (as reported in Figure 1)	n	Operational criterion applied at full-text assessment	Typical profile of records excluded under this category
Fluorescence-only indocyanine green (ICG) imaging, outside revised scope	**31**	Record excluded if near-infrared fluorescence (ICG or equivalent dye-based fluorescence) was the sole visualization or guidance modality reported, and the record contained no augmented-reality overlay, no mixed-reality visualization, no computer-vision or artificial-intelligence image analysis, and no three-dimensional model or virtual-reality reconstruction. Records combining fluorescence with any of these four components were retained and assessed on their AR/MR/CV/3D content. ICG terminology was deliberately retained in the search strings in order to retrieve such hybrid records (Section 2.5).	Clinical and feasibility reports of ICG-guided perfusion assessment, ureteric or vascular delineation, and lesion delineation in gynecologic laparoscopy, in which the technology under evaluation was the fluorescence agent and imaging platform itself rather than any computational or model-based guidance layer.
Gynecologic oncology-only or sentinel lymph node studies	**8**	Record excluded if the entire study population and all reported operative objectives concerned malignant gynecologic disease or sentinel lymph node mapping, with no benign, uterus-preserving, or fertility-preserving indication reported and no extractable subgroup relevant to the review population defined in Section 2.2. Oncological records reporting a separable benign or fertility-sparing subgroup were retained.	Sentinel-node mapping and lymphatic-navigation studies in cervical or endometrial carcinoma; AR or 3D navigation studies in radical hysterectomy, pelvic exenteration, or nodal staging, where the operative goal was oncological clearance rather than preservation of reproductive anatomy.
Reviews, editorials, and commentary without usable primary data	**7**	Record excluded if it was a narrative review, systematic review, editorial, commentary, viewpoint, or invited perspective that reported no primary clinical, technical, dataset, or experimental result attributable to that record. Reference lists of these records were nevertheless screened for citation searching (Section 2.3); no additional unique record was identified.	Narrative overviews of digital and image-guided technologies in gynecologic surgery, editorial commentary accompanying primary articles, and review articles restating findings already captured through the included primary records.

Exclusion category (as reported in Figure 1)	n	Operational criterion applied at full-text assessment	Typical profile of records excluded under this category
Education-, training-, or simulation-only augmented reality	**4**	Record excluded if the reported application of AR, MR, or 3D modeling was confined to teaching, resident or student training, skills assessment, simulation-based rehearsal outside patient care, or patient counselling and education, with no application to intraoperative guidance, operative decision-making, or preoperative planning for an actual planned procedure.	AR- or VR-based laparoscopic skills trainers and curricula; simulation-based competency assessment; 3D-printed or virtual models produced solely for anatomy teaching or for patient consent and counselling.
Diagnostic-only imaging without surgical-planning application	**4**	Record excluded if the reported computational or three-dimensional imaging output was directed exclusively at diagnosis, disease detection, staging, or radiological characterization, and the record reported no application of that output to operative planning, intraoperative orientation, registration, navigation, or surgical decision-making.	Machine-learning and radiomic models for the detection or classification of adenomyosis, deep endometriosis, or uterine pathology on MRI or ultrasound, evaluated against radiological or histological reference standards without any reported surgical-planning or intraoperative use.
Failure of one or more mandatory eligibility parameters (previously labelled “insufficiently direct to the review question”)	**2**	This category was applied where a record satisfied the technology criterion but failed at least one of four mandatory eligibility parameters, each stated below as a question answerable from the record itself. A record was excluded under this category if the answer to any one of P1 to P4 was “no”. Both records excluded under this category failed P1. **P1 — Population.** Does the record report a procedure performed on the female reproductive tract, and is that procedure uterus-preserving or otherwise anatomy-preserving? Records reporting non-gynecologic pelvic or abdominal procedures fail P1 unless a gynecologic subgroup or a gynecologic application is separately reported. For enabling-technology records (Stream B, Section 3.2), P1 is satisfied where the anatomical target, the imaging source, or the operative setting is gynecologic, irrespective of whether the index procedure was itself uterus-preserving; a computer-vision development study conducted during total laparoscopic hysterectomy [17] therefore satisfies P1, whereas a record reporting a clinical outcome of hysterectomy would not.<br>**P2 — Technology.** Is the augmented-reality, mixed-reality, computer-vision, artificial-intelligence, or three-dimensional modeling component described in sufficient detail to be assigned to one of the categories in Table 1, and is it either applied or evaluated within the record? A record fails P2 if the technology is named only in the introduction, discussion,	Both records excluded under this category failed P1. They reported image guidance or three-dimensional modeling applied to non-gynecologic pelvic or abdominal procedures, with no gynecologic subgroup, no gynecologic application, and no separately reported result from which a gynecologic inference could be drawn.

Exclusion category (as reported in Figure 1)	n	Operational criterion applied at full-text assessment	Typical profile of records excluded under this category
		or future-directions section without being described, applied, or evaluated in the work reported.<br>**P3 — Context.** Is the technology applied to surgical planning, intraoperative guidance, intraoperative orientation or navigation, or operative decision-making? A record fails P3 if the technology is directed exclusively to diagnosis, education, training, patient counselling, or postoperative assessment with no surgical application reported.<br>**P4 — Primary data.** Does the record report at least one primary result of its own — clinical, technical, dataset, or experimental — attributable to that record? A record fails P4 if it reports only findings previously published elsewhere.<br>Relationship to the other exclusion categories. Parameters P1 to P4 are not exclusive to this category; they are the eligibility parameters applied to every retrieved full text (Section 2.5). Where a record failed a parameter for a reason already named by one of the five preceding categories — oncology-only (P1), education- or training-only (P3), diagnostic-only (P3), or review without primary data (P4) — it was assigned to that more specific category. The present category was used only for records that failed a parameter for a reason not captured by any of those five, and is therefore residual by construction rather than by absence of definition. It is the smallest category in the review, comprising two of the 56 excluded full texts.	
**Total full-text records excluded**	**56**	—	—

Panel B. Record flow across all screening stages

Screening stage	n	Basis of decision
Records identified (three sources)	**528**	PubMed/MEDLINE n = 242; Scopus n = 132; Google Scholar n = 154 (Section 2.3; search executed 11 February 2026, confirmatory re-run 16 May 2026).
Duplicates removed before screening	**92**	Records retrieved by more than one source, identified by matching digital object identifier, then by title and first-author concordance.
Excluded at title and abstract	**360**	Records in which the title and abstract established that no AR, MR, computer-vision/AI, or 3D-modeling component was present, or that the clinical context was

Screening stage	n	Basis of decision
		outside benign gynecologic surgery. Reasons at this stage were not recorded by category, in accordance with PRISMA-ScR item 14, which requires reasons for exclusion to be reported at the stages at which eligibility is formally assessed.
Full texts sought and retrieved	**76**	All records sought were retrieved; none was unobtainable.
Excluded at full text	**56**	By category, as set out in Panel A above.
Included in the qualitative synthesis	**20**	Charted in Tables 2–4 and appraised in Table 5.

Note on the granularity of the exclusion record

Reasons for exclusion were documented prospectively at the level of exclusion category rather than at the level of the individual bibliographic record, and a record-level log of the 56 excluded full texts was not retained. This table therefore reports the operational criterion and the count for each category, but does not list the excluded records individually. PRISMA-ScR item 14 requires that the numbers of sources screened, assessed for eligibility, and included be reported together with the reasons for exclusion at each stage, ideally in a flow diagram; the present table and Figure 1 together satisfy that requirement at category level. The authors recognize that a record-level list would permit independent re-audit of every individual selection decision, and prospective retention of such a log has been adopted for subsequent reviews by this group.

Abbreviations: AI, artificial intelligence; AR, augmented reality; CV, computer vision; ICG, indocyanine green; MR, mixed reality; MRI, magnetic resonance imaging; PRISMA-ScR, Preferred Reporting Items for Systematic Reviews and Meta-Analyses extension for Scoping Reviews; VR, virtual reality; 3D, three-dimensional.
