# Supplementary material for: Augmented Reality, Mixed Reality, Computer Vision, and Three-Dimensional Modeling in Fertility-Preserving Minimally Invasive Gynecologic Surgery: A Scoping Review with Narrative Synthesis
Source: Medicina (Kaunas). 2026 Sep 17;62(9):1790. doi: 10.3390/medicina62091790 (PMC13609672; doi:10.3390/medicina62091790)
Supplement: Supplementary file 1 [file medicina-62-01790-s001.zip › Table_S3_Critical_Appraisal.pdf]

Supplementary Material – Table S3

Study-Level Critical Appraisal, Cross-Design Domain Grid, and Record-to-Table Correspondence

Manuscript: "Augmented Reality, Mixed Reality, Computer Vision, and Three-Dimensional Modeling in Fertility-Preserving Minimally Invasive Gynecologic Surgery: A Scoping Review with Narrative Synthesis" (medicina-4409602).

Panel A. Study-level appraisal of all 20 included records

Because the included records span four fundamentally different designs, no single risk-of-bias instrument applies to all of them. Each record was therefore appraised with the instrument appropriate to its design, as stated in the third column. The judgement reached in each domain is given in full, together with the basis for that judgement, so that a reader can disagree with a specific judgement on stated grounds rather than having to accept an unexplained summary rating. Domain labels D1–D6 correspond to the cross-design domains defined in Panel B.

Included record and evidence stream	Design as reported	Appraisal instrument applied	Domain judgements and basis	Interpretation for this review
Bourdel et al. 2017 [3] Stream B	Experimental user study; 10 residents; physical uterine model	Structured feasibility appraisal	D1 high: participants were residents, not patients, and the target was a physical model. D2 low: an internal comparator (AR versus MRI-only localization) was used. D3 high: experimental bench setting. D4 moderate: localization accuracy is semi-objective. D5 not applicable. D6 not applicable.	Proof of concept. No inference to patient care is possible, but the internal comparison is a genuine methodological strength for a bench study.
Bourdel et al. 2017 [4] Stream A	Clinical feasibility report; 3 patients; laparoscopic myomectomy	JBI case-series principles	D1 high: three patients, selection process not described. D2 high: no comparator. D3 low: the intended population and procedure. D4 high: outcome is descriptive feasibility, no predefined measure. D5 moderate: perioperative course reported, follow-up duration not specified. D6 high: no fertility outcome.	Establishes that the workflow can be executed intraoperatively in patients. No effect estimate is possible.
Bourdel et al. 2019 [6] Stream A	Case report; 2 patients; adenomyomectomy	JBI case-report principles	D1 high: two cases. D2 high: no comparator. D3 low: adenomyomectomy is directly within scope. D4 high: narrative outcome. D5 unclear: follow-up not stated. D6 high: no fertility outcome.	Feasibility in the setting where endometrial-cavity awareness matters most. Not generalizable.
Chauvet et al. 2020 [5] Stream A	Case reports; 2 patients; laparoscopic myomectomy with diffusion tensor imaging	JBI case-report principles	D1 high: two cases. D2 high: no comparator. D3 low: within scope. D4 high: the stated rationale (uterine scar quality) was not measured. D5 unclear: no obstetric or scar follow-up. D6 high: no fertility outcome.	Technically novel visualization of myometrial fibre orientation. The clinical claim regarding scar quality remains an untested hypothesis.
Akladios et al. 2020 [18] Stream B	Animal model; 5 porcine subjects; 58 surgeon video assessments	Structured feasibility appraisal	D1 high: porcine anatomy. D2 low: AR compared with direct vision. D3 high: non-gynecologic target structure (ureter), animal setting. D4 moderate: multi-rater but unblinded subjective assessment. D5 moderate. D6 not applicable.	Addresses a real technical problem, localization of a hidden structure, with an unusually large rater panel. Indirect for fertility-preserving human surgery.
Ochi et al. 2023 [19] Stream A	Case report; 1 patient;	JBI case-report principles	D1 high: single case. D2 high: no comparator. D3 low: within scope. D4 high: no defined	Demonstrates that a commercial mixed-reality stack can be deployed

Included record and evidence stream	Design as reported	Appraisal instrument applied	Domain judgements and basis	Interpretation for this review
	laparoscopic myomectomy with mixed reality		outcome set. D5 moderate: uneventful course reported. D6 high: no fertility outcome.	in a live gynecologic case. No effect evidence.
Torabinia et al. 2022 [20] Stream B	Ex-vivo feasibility; single uterine fibroid model	Structured feasibility appraisal	D1 high: one specimen. D2 moderate: compared against two-dimensional monitor review. D3 high: ex-vivo. D4 high: outcome is the operator's perceived spatial visualization. D5 not applicable. D6 not applicable.	A human-factors signal only. The comparison is internal and unblinded.
Comptour et al. 2025 [21] Stream A	Retrospective matched case-control; 34 patients (17 AR, 17 control); myomectomy and adenomyomectomy	ROBINS-I domains	Confounding: serious — 1:1 optimal pair matching on lesion type, size, and FIGO location reduces but does not remove selection by surgeon preference and case complexity, and no multivariable adjustment was performed. Selection of participants: moderate — all 17 available AR cases were used and 17 controls were selected from a pool of 40 over May 2016 to September 2021. Classification of intervention: low. Deviations from intended intervention: low. Missing data: moderate. Outcome measurement: low — the primary endpoint (non-inferiority of operative time, margin 15 minutes) and complications are objectively recorded. Selective reporting: moderate — desire for and number of pregnancies were prespecified data-collection items but pregnancy was not among the stated primary or secondary endpoints. Overall: serious risk of bias. D6 moderate rather than high: this is the only included record to report any reproductive outcome (median follow-up 18 months; 6 of 11 AR patients with pregnancy desire conceived versus 5 of 12 controls, $p = 1.00$), but the study was powered for operative time and no live birth, miscarriage, uterine-rupture, or obstetric outcome was reported.	The most clinically informative AR record in this review, and the only source of reproductive data in the entire included evidence. Supports the absence of added harm and non-inferior operative time. The pregnancy comparison is descriptive only: six versus five events in 34 retrospectively identified patients cannot support inference in either direction.
Collins et al. 2021 [22] Stream B	Technical development; sample size not reported	Structured feasibility appraisal	D1 unclear: sample not reported in the source. D2 high: no clinical comparator. D3 high: algorithmic evidence. D4 moderate: registration performance reported technically. D5 unclear. D6 not applicable.	Addresses markerless registration and tracking of the mobile uterus, a genuine prerequisite for intraoperative AR. Not clinical evidence.
François et al. 2020 [23] Stream A	Technical study with intraoperative user evaluation; 3,818 labelled images; 10 gynecologic laparoscopies; 5 surgeons	Structured feasibility appraisal	D1 moderate: multi-surgeon evaluation, dataset adequate for the task. D2 low: internal comparator for surgeon interaction time and registration accuracy. D3 moderate: enabling technology, but evaluated during live surgery. D4 low: interaction time and registration accuracy are objective. D5 moderate. D6 not applicable.	Methodologically the strongest enabling-technology record in the review, and the only one evaluated during live human surgery. Reports no patient outcome.

Included record and evidence stream	Design as reported	Appraisal instrument applied	Domain judgements and basis	Interpretation for this review
Prokopetc et al. 2015 [24] Stream B	Technical study; 95 images	Structured feasibility appraisal	D1 high: small image set. D2 moderate: internal reference annotation. D3 high: technical only. D4 moderate. D5 unclear. D6 not applicable.	An early technical contribution, superseded in scale by later datasets in this review.
Sato et al. 2019 [17] Stream B	Preliminary video study; 19 total laparoscopic hysterectomy cases	Structured feasibility appraisal	D1 high: convenience video series. D2 high: no comparator. D3 high: the procedure is total laparoscopic hysterectomy, which is not fertility-preserving. D4 high: ureter detection reported by the authors as unsatisfactory. D5 moderate. D6 not applicable.	Retained for completeness of the technical map. This record has the lowest directness to the review question of any included study.
Madad Zadeh et al. 2023 [25] Stream B	Dataset and annotation study; 3,800 images	Structured feasibility appraisal	D1 moderate: dataset size adequate. D2 not applicable. D3 high: infrastructure rather than application. D4 moderate: annotation protocol reported, inter-annotator agreement not assessed in the source. D5 unclear. D6 not applicable.	Provides infrastructure for future AR automation. No clinical or performance claim is evaluated here.
Netter et al. 2025 [7] Stream B	Multicentre proof-of-concept; 112 operative videos; YOLOv5 architecture	Structured feasibility appraisal	D1 moderate: multicentre sourcing is a strength. D2 moderate: expert annotation as reference standard. D3 moderate: endometriosis laparoscopy is within scope, but analysis was retrospective and offline. D4 moderate: performance reported as variable across lesion classes. D5 moderate. D6 not applicable.	The most clinically proximate lesion-recognition record in the review. Does not constitute real-time intraoperative navigation.
Leibetseder et al. 2022 [8] Stream B	Technical artificial-intelligence study; operative video dataset	Structured feasibility appraisal	D1 moderate. D2 moderate: two architectures compared against annotation. D3 high: no clinical deployment. D4 moderate: moderate reported performance. D5 unclear. D6 not applicable.	A benchmarking contribution for lesion detection and localization.
Flaxman et al. 2024 [26] Stream A	Surgical planning evaluation; 7 complex multifibroid uterus cases, including 5 myomectomies	JBI case-series and structured surgeon-assessment appraisal	D1 high: seven cases selected for complexity, of which two were hysterectomies rather than myomectomies, so only five procedures were uterus-preserving. D2 high: no comparator. D3 moderate: planning application within scope, but part of the sample is not fertility-preserving surgery. D4 high: the primary outcome is surgeon-reported change of dissection route, collected unblinded; the reported denominator of 15 is at the level of the surgeon–case response, five surgeons (two staff and three fellows) having returned one or two assessments per case, and the questionnaire is not reported to have been validated. D5 moderate. D6 high: no fertility follow-up.	Supports planning utility as perceived by the operating surgeons, in a mixed sample that is only partly uterus-preserving. Provides no outcome effect estimate.
Li et al. 2026 [27] Stream A	Single-centre randomized controlled trial; 133 randomized and analysed by intention to treat, 110 in the per-	Randomized-trial risk-of-bias domains	Randomization process: low — computer-generated sequence, 1:1 allocation, variable blocks of 4, 6, and 8, stratified by fibroid number. Allocation concealment: low — sequentially numbered, opaque, sealed envelopes prepared by an independent statistician. Blinding of participants and	The only Level II record in the review, and methodologically stronger than the previously submitted appraisal indicated: allocation concealment, complication-assessor blinding, and handling of missing data are all

Included record and evidence stream	Design as reported	Appraisal instrument applied	Domain judgements and basis	Interpretation for this review
	protocol sensitivity analysis (55 per group); single-port laparoscopic multiple myomectomy		personnel: high — neither patients nor surgeons could be blinded to the presence of a physical model. Blinding of outcome assessment: low for complications — assessors of postoperative complications were blinded to allocation, and analysts were blinded until the analysis was complete; blinding for other outcomes is not stated. Missing outcome data: low — all 133 randomized patients were included in the primary intention-to-treat analysis with multiple imputation by chained equations, and the 110-patient per-protocol population was a prespecified sensitivity analysis. Selective reporting: moderate — two co-primary endpoints (operative time and estimated blood loss) were declared without a stated multiplicity adjustment, and trial registration (ChiCTR2500112904, November 2025) post-dated completion of enrolment (March to November 2024) by approximately twelve months. Fertility outcomes: not reported, although desire for fertility preservation was an inclusion criterion. Overall: some concerns, driven by the impossibility of blinding and by retrospective registration.	adequately reported. Demonstrates an operative-process benefit of preoperative 3D printing — not any reproductive benefit — and concerns 3D printing rather than intraoperative AR.
Martel et al. 2026 [9] Stream A	Retrospective feasibility and expert evaluation; 14 models; colorectal endometriosis	Structured feasibility appraisal	D1 high: retrospective, single centre. D2 high: no comparator. D3 moderate: planning application within scope. D4 high: outcome is expert perception of usefulness. D5 unclear. D6 high: no fertility outcome.	Perceptual planning value in an expert cohort. No comparative or outcome inference.
Borghese et al. 2022 [10] Stream A	Prospective pilot cohort; 7 women; rectosigmoid endometriosis	JBI case-series principles	D1 moderate: prospective recruitment is a strength. D2 moderate: the operation itself served as an internal reference standard. D3 moderate. D4 high: concordance was assessed by the operating team without blinding. D5 moderate. D6 high: no fertility outcome.	Demonstrates concordance between the model and the operative anatomy. Concordance is not clinical benefit.
Zhang et al. 2026 [11] Stream A	Composite design: retrospective feasibility (147 screened, 41 reconstructable), expert evaluation (2 surgeons), national survey (31 respondents), and preliminary prospective evaluation (2 patients)	Structured feasibility appraisal	D1 moderate: component denominators are stated (147 patients screened retrospectively, of whom 41 had MRI datasets permitting reconstruction; 31 survey respondents; 2 expert surgeons; 2 prospectively evaluated patients), although no single overall sample size is given and the components cannot be summed. D2 high: no comparator. D3 moderate. D4 high: survey and perception outcomes. D5 moderate: each component is reported, but across four heterogeneous components with different participant types. D6 high: no fertility outcome.	Exploratory, and the prospective clinical component comprises two patients. Useful as a feasibility and clinician-acceptability signal only.

Abbreviations: AR, augmented reality; JBI, Joanna Briggs Institute; MR, mixed reality; ROBINS-I, Risk Of Bias In Non-randomized Studies of Interventions; 3D, three-dimensional.

Panel B. Cross-design domain grid

The four design-specific instruments used in Panel A produce judgements that are not directly comparable with one another. To permit a single comparable view across all 20 records, six domains were defined that can be evaluated for every design in this review, whether clinical, technical, or experimental. The grid is color-coded so that the distribution of concerns across records and domains can be read at a glance. It is a summary of applicability and of the principal bias concerns; it is not a substitute for the design-specific appraisal in Panel A, and it does not constitute a certainty-of-evidence assessment.

Domain definitions. D1, selection and representativeness: whether the participants, specimens, images, or videos studied represent the population or material to which the finding is intended to apply. D2, comparator or reference standard: whether an appropriate internal comparator or reference standard was used. D3, directness to the review question: how closely the population, technology, and clinical context correspond to the population–concept–context framework of this review (Section 2.2). D4, objectivity of outcome measurement: whether the primary outcome was measured objectively or consisted of unblinded operator perception. D5, completeness of outcome reporting and follow-up: whether outcomes, denominators, and follow-up were reported completely. D6, reporting of fertility-relevant outcomes: whether any fertility, pregnancy, obstetric, or long-term reproductive outcome was reported. D6 is recorded as not applicable for records in which no patient was treated, since a fertility outcome could not arise; it is recorded as high concern for records in which patients were treated in a fertility-preserving context and no reproductive outcome was reported, and as moderate concern where a reproductive outcome was reported but was not a stated endpoint and the study was not powered for it.

Included record	Stream	D1 Selection and representativeness	D2 Comparator or reference standard	D3 Directness to the review question	D4 Objectivity of outcome measurement	D5 Completeness of reporting and follow-up	D6 Reporting of fertility-relevant outcomes
Bourdel et al. 2017 [3]	**B**	High	Low	High	Moderate	NA	NA
Bourdel et al. 2017 [4]	**A**	High	High	Low	High	Moderate	High
Bourdel et al. 2019 [6]	**A**	High	High	Low	High	Unclear	High
Chauvet et al. 2020 [5]	**A**	High	High	Low	High	Unclear	High
Akladios et al. 2020 [18]	**B**	High	Low	High	Moderate	Moderate	NA
Ochi et al. 2023 [19]	**A**	High	High	Low	High	Moderate	High
Torabinia et al. 2022 [20]	**B**	High	Moderate	High	High	NA	NA
Comptour et al. 2025 [21]	**A**	Moderate	Low	Low	Low	Moderate	Moderate
Collins et al. 2021 [22]	**B**	Unclear	High	High	Moderate	Unclear	NA
François et al. 2020 [23]	**A**	Moderate	Low	Moderate	Low	Moderate	NA
Prokopetc et al. 2015 [24]	**B**	High	Moderate	High	Moderate	Unclear	NA
Sato et al. 2019 [17]	**B**	High	High	High	High	Moderate	NA
Madad Zadeh et al. 2023 [25]	**B**	Moderate	NA	High	Moderate	Unclear	NA
Netter et al. 2025 [7]	**B**	Moderate	Moderate	Moderate	Moderate	Moderate	NA

Included record	Stream	D1 Selection and representativeness	D2 Comparator or reference standard	D3 Directness to the review question	D4 Objectivity of outcome measurement	D5 Completeness of reporting and follow-up	D6 Reporting of fertility-relevant outcomes
Leibetseder et al. 2022 [8]	**B**	Moderate	Moderate	High	Moderate	Unclear	NA
Flaxman et al. 2024 [26]	**A**	High	High	Moderate	High	Moderate	High
Li et al. 2026 [27]	**A**	Low	Low	Moderate	Moderate	Low	High
Martel et al. 2026 [9]	**A**	High	High	Moderate	High	Unclear	High
Borghese et al. 2022 [10]	**A**	Moderate	Moderate	Moderate	High	Moderate	High
Zhang et al. 2026 [11]	**A**	Moderate	High	Moderate	High	Moderate	High

NA, not applicable to this design. Cell shading indicates the rating: green, low concern; amber, moderate concern; red, high concern; grey, unclear from the published report; unshaded, not applicable to that design. Two patterns should be noted. First, D4 is the most frequently compromised domain, because the dominant outcome across this literature is the operating surgeon's own perception of usefulness, recorded without blinding. Second, D6 is rated high concern in every record in which patients were treated, with a single exception: the matched AR series [21] is the only included record to report any reproductive outcome, and is rated moderate rather than high on that basis. No record in the review — including the single randomized controlled trial — reports live birth, miscarriage, uterine rupture, postoperative uterine integrity, or any obstetric outcome, so the absence of reproductive evidence remains a property of the evidence base as a whole rather than of any individual study.

Panel C. Correspondence between the included records and the evidence tables

This panel demonstrates that the 20 records counted in the qualitative synthesis are the same 20 records presented in the evidence tables, and shows for each record its technology category, evidence stream, evidence level, and every table in which it appears. Tables 3 and 4 are procedure-focused and topic-focused subsets of Table 2 and are not expected to contain every record; Tables 2 and 5 are expected to contain all 20.

Included record	Technology category (Table 1)	Procedure or context	Stream	Evidence level	Table 2	Table 3	Table 4	Table 5
Bourdel et al. 2017 [3]	AR overlay	Myomectomy model	B	V	Yes	Yes	—	Yes*
Bourdel et al. 2017 [4]	AR overlay	Laparoscopic myomectomy	A	V	Yes	Yes	—	Yes
Bourdel et al. 2019 [6]	AR overlay	Adenomyomectomy	A	V	Yes	Yes	—	Yes
Chauvet et al. 2020 [5]	AR overlay with DTI	Laparoscopic myomectomy	A	V	Yes	Yes	—	Yes
Akladios et al. 2020 [18]	AR overlay	Ureter localization	B	V	Yes	—	—	Yes
Ochi et al. 2023 [19]	MR visualization	Laparoscopic myomectomy	A	V	Yes	Yes	—	Yes
Torabinia et al. 2022 [20]	MR visualization	Mock myomectomy	B	V	Yes	Yes	—	Yes
Comptour et al. 2025 [21]	AR overlay	Myomectomy / adenomyomectomy	A	III	Yes	Yes	—	Yes
Collins et al. 2021 [22]	AR registration and tracking	Uterine laparoscopy	B	V	Yes	—	—	Yes
François et al. 2020 [23]	AI / computer vision	Uterine contour detection	A	V	Yes	—	Yes	Yes
Prokopetc et al. 2015 [24]	AI / computer vision	Uterus and FU-junction detection	B	V	Yes	—	Yes	Yes
Sato et al. 2019 [17]	Computer vision	Total laparoscopic hysterectomy	B	V	Yes	—	Yes	Yes
Madad Zadeh et al. 2023 [25]	AI / computer vision dataset	Gynecologic laparoscopy	B	V	Yes	—	Yes	Yes
Netter et al. 2025 [7]	AI lesion recognition	Endometriosis laparoscopy	B	V	Yes	—	Yes	Yes
Leibetseder et al. 2022 [8]	AI lesion localization	Endometriosis laparoscopy	B	V	Yes	—	Yes	Yes
Flaxman et al. 2024 [26]	3D printing	Multifibroid uterus	A	IV	Yes	Yes	—	Yes
Li et al. 2026 [27]	3D printing	Single-port multiple myomectomy	A	II	Yes	Yes	—	Yes

Included record	Technology category (Table 1)	Procedure or context	Stream	Evidence level	Table 2	Table 3	Table 4	Table 5
Martel et al. 2026 [9]	3D / VR modeling	Colorectal endometriosis	A	IV	Yes	—	Yes	Yes
Borghese et al. 2022 [10]	3D virtual modeling	Rectosigmoid endometriosis	A	IV	Yes	—	Yes	Yes
Zhang et al. 2026 [11]	3D modeling	Deep endometriosis	A	IV	Yes	—	Yes	Yes

* In the previously submitted version, Bourdel et al. 2017 [3] was the only included record absent from the appraisal in Table 5. This omission was identified during the present revision through the correspondence check reported here, and Table 5 has been amended so that all 20 records now appear in the appraisal. Tables 2 and 5 each now contain all 20 records; Table 3 contains the 9 myomectomy and adenomyomectomy records; Table 4 contains the 9 endometriosis-related and enabling computer-vision records. Abbreviations: AI, artificial intelligence; AR, augmented reality; DTI, diffusion tensor imaging; FU, fallopian tube-uterus; MR, mixed reality; VR, virtual reality; 3D, three-dimensional.
