# Supplementary material for: Augmented Reality, Mixed Reality, Computer Vision, and Three-Dimensional Modeling in Fertility-Preserving Minimally Invasive Gynecologic Surgery: A Scoping Review with Narrative Synthesis
Source: Medicina (Kaunas). 2026 Sep 17;62(9):1790. doi: 10.3390/medicina62091790 (PMC13609672; doi:10.3390/medicina62091790)
Supplement: Supplementary file 1 [file medicina-62-01790-s001.zip › Table_S4_Search_Strategies_updated.pdf]

## Supplementary Material – Table S4

### Complete Search Strategies as Executed

*Manuscript: "Augmented Reality, Mixed Reality, Computer Vision, and Three-Dimensional Modeling in Fertility-Preserving Minimally Invasive Gynecologic Surgery: A Scoping Review with Narrative Synthesis" (medicina-4409602).*

Primary search executed 11 February 2026. Confirmatory re-run of the identical strategies executed 16 May 2026, which retrieved no additional eligible record. Coverage: database inception to the search date.

### Panel A. Sources, structure of the search, and yield

Two conceptual domains were searched in every source: visualization or computing technology, and gynecologic procedure or benign uterine and endometriotic pathology. The way those domains were expressed differs between sources, because the platforms implement different field-tagging and limiter conventions and an identical literal string would have been syntactically invalid on at least two of the three platforms. In PubMed/MEDLINE the domains were expressed as three blocks combined with AND, with a closing NOT clause to remove animal-only records. In Scopus they were expressed as five focused TITLE-ABS-KEY concept pairs combined with OR, each pair pre-combining a technology term with a procedure or pathology term. In Google Scholar, which supports neither field tags nor truncation, they were expressed as seven bounded phrase pairs. The strings in Panels B to D are reproduced exactly as executed.

| Source | Syntax and limiters available on the platform | Structure of the search, and notes | Records retrieved |
|---|---|---|---|
| **1. PubMed/MEDLINE** | Field tags [Title/Abstract] and [MeSH Terms]; truncation with *; English-language and human filters applied through the interface; preprints excluded through the interface. | Three concept blocks combined with AND: visualization or computing technology, gynecologic procedure, and benign uterine or endometriotic pathology. A closing NOT clause removed animal-only records. The string as executed is reproduced verbatim in Panel B. | **242** |
| **2. Scopus** | TITLE-ABS-KEY() field code; Boolean operators; document-type limiters (conference papers, books, book chapters, letters, editorials, notes, errata, and short surveys excluded); English-language limiter. | Scopus does not accept the PubMed field-tag syntax, and MeSH vocabulary is not implemented in Scopus. The search was therefore built as five focused TITLE-ABS-KEY concept pairs combined with OR, each pair pre-combining a technology term with a procedure or pathology term. The string as executed is reproduced verbatim in Panel C. | **132** |
| **3. Google Scholar** | No field tags and no truncation supported; bounded phrase pairs used; relevance ranking; private browser session; first 25 results screened per query. | Seven focused phrase-pair queries were executed, listed in Panel D. This source was used as a supplementary coverage safeguard and contributed no unique included record (Section 2.3). | **154** |

### Panel B. PubMed/MEDLINE string, as executed

(("augmented reality"[Title/Abstract] OR "mixed reality"[Title/Abstract] OR
"image-guided"[Title/Abstract] OR "image guided"[Title/Abstract] OR
"computer-assisted"[Title/Abstract] OR "computer assisted"[Title/Abstract] OR
"computer vision"[Title/Abstract] OR "artificial intelligence"[Title/Abstract] OR
"deep learning"[Title/Abstract] OR "three-dimensional model*"[Title/Abstract] OR
"3D model*"[Title/Abstract] OR "indocyanine green"[Title/Abstract])
AND
("gynecologic surgery"[Title/Abstract] OR "gynaecologic surgery"[Title/Abstract] OR
gynecology[Title/Abstract] OR gynaecology[Title/Abstract] OR laparoscopy[Title/Abstract] OR
laparoscopic[Title/Abstract] OR robotic[Title/Abstract])
AND
(myomectomy[Title/Abstract] OR myoma*[Title/Abstract] OR fibroid*[Title/Abstract] OR
leiomyoma*[Title/Abstract] OR adenomyosis[Title/Abstract] OR adenomyomectomy[Title/Abstract] OR
endometriosis[Title/Abstract] OR "deep endometriosis"[Title/Abstract] OR
"deep infiltrating endometriosis"[Title/Abstract] OR ureter*[Title/Abstract] OR
fertility-preserving[Title/Abstract] OR reproductive[Title/Abstract]))
NOT
(animals[MeSH Terms] NOT humans[MeSH Terms])

Interface limiters applied: English language; Humans; preprints excluded. Records retrieved: 242.

## Panel C. Scopus string, as executed

The five concept pairs below were run as a single combined advanced search and returned 132 English-language records after document-type exclusions.

TITLE-ABS-KEY("augmented reality" AND gynecolog*)
OR
TITLE-ABS-KEY("augmented reality" AND myomectomy)
OR
TITLE-ABS-KEY("mixed reality" AND myomectomy)
OR
TITLE-ABS-KEY(("3D model" OR "three-dimensional model" OR "virtual reality") AND endometriosis)
OR
TITLE-ABS-KEY(("3D model" OR "three-dimensional model" OR "3D printed" OR "three-dimensional printed") AND (fibroid OR fibroids OR myoma OR myomas OR leiomyoma OR leiomyomas OR myomectomy))

Limiters applied: English language; conference papers, books, book chapters, letters, editorials, notes, errata, and short surveys excluded where applicable. Records retrieved: 132.

## Panel D. Google Scholar queries, as executed

Seven focused queries, screened by relevance ranking to a maximum depth of 25 records per query, in a private browser session:

"augmented reality" "gynecologic surgery"
"augmented reality" "laparoscopic myomectomy"
"augmented reality" "adenomyoma"
"mixed reality" "laparoscopic myomectomy"
"augmented laparoscopy" uterus contour detection
"computer vision" "gynecologic laparoscopy"
"3D model" "deep endometriosis" "surgical planning"

Records screened: 154. Unique included records contributed by this source: none. Every included study was independently retrieved by the PubMed/MEDLINE or Scopus search.

## Panel E. Citation searching

Reference lists of all included articles and of relevant reviews identified during screening were examined. No additional unique eligible record was identified through citation searching.

*Note. Changes introduced in the present revision are shown in red. The complete strategies were moved from the main text to this supplementary table at the editor's request; Section 2.3 of the manuscript now carries a concise description of the sources, the search period, and the overall search approach.*
