# Supplementary material for: Augmented Reality, Mixed Reality, Computer Vision, and Three-Dimensional Modeling in Fertility-Preserving Minimally Invasive Gynecologic Surgery: A Scoping Review with Narrative Synthesis
Source: Medicina (Kaunas). 2026 Sep 17;62(9):1790. doi: 10.3390/medicina62091790 (PMC13609672; doi:10.3390/medicina62091790)
Supplement: Supplementary file 1 [file medicina-62-01790-s001.zip › Table_S5_Implementation.pdf]

Supplementary Material – Table S5

Implementation Characteristics of the Included Studies, and Completeness of Technical Reporting

Manuscript: "Augmented Reality, Mixed Reality, Computer Vision, and Three-Dimensional Modeling in Fertility-Preserving Minimally Invasive Gynecologic Surgery: A Scoping Review with Narrative Synthesis" (medicina-4409602).

This table charts the information a centre would need in order to reproduce or adopt each workflow. Only information stated in the published report was charted; no field was inferred from the manufacturer, from a related publication by the same group, or from the technology's general characteristics. Where the field could not be established from the report it is recorded as NR (not reported), and where it does not apply to the design it is recorded as not applicable. The resulting pattern is itself a finding of this review rather than a limitation of the charting process. Source imaging modality is reported by every record, and the registration or alignment approach is described in principle by most records that have one. Two gaps are systematic. First, a quantitative accuracy metric is reported by only five of the twenty records; for the remainder the reader is told how registration was attempted but not how well it performed. Second, the display or head-mounted hardware and the specific software platform and version are frequently unnamed, which prevents a reader from establishing what was actually used, still less procuring it. A described registration method without a measured accuracy cannot be assessed for fitness in fertility-preserving surgery, where the tolerance for misregistration is set by the proximity of the endometrial cavity and the ureter; and an unnamed platform cannot be reproduced. For most of the workflows in this review, including several applied in patients, published reporting is therefore insufficient to permit independent technical replication or informed adoption.

Included record	Software or segmentation platform	Display or hardware	Source imaging modality	Registration or alignment method	Quantitative accuracy reported
Bourdel et al. 2017 [3]	In-house AR software; uterus and myomas segmented manually from MRI by a radiologist; segmentation platform NR	Standard PC with GPU; Karl Storz 10-mm HD laparoscope; pelvic trainer; no head-mounted display	MRI of the uterine model (sagittal, axial, and coronal series)	Automatic real-time model-to-video fusion using a two-phase wide-baseline multi-texturemap registration	Yes — mean localization error 0.64 mm (0.01–4.71) with AR versus 16.80 mm (0.1–52.2) without
Bourdel et al. 2017 [4]	Medical Imaging Interaction Toolkit (MITK) for interactive segmentation; the authors' own AR software, not named	Standard laparoscopic stack with 0° Karl Storz Spies laparoscope; monitor overlay; no head-mounted display	Preoperative T2-weighted MRI	Dense structure-from-motion reconstruction, then semi-automatic preoperative-to-intraoperative registration requiring manual marking of organ-boundary contours, then real-time tracking and fusion	No
Bourdel et al. 2019 [6]	NR	Laparoscopic monitor overlay	MRI (T2-weighted)	NR	No
Chauvet et al. 2020 [5]	NR	Laparoscopic monitor overlay	MRI with diffusion tensor imaging and fibre tractography	NR	No
Akladios et al. 2020 [18]	LASAR; separate segmentation platform NR	NR	Intraoperative cone-beam computed tomography following ureteral contrast administration	NR	Yes — ureter localization margin of error 1.77 ± 1.56 mm
Ochi et al. 2023 [19]	Holoeyes XR; fibroids delineated by a region-	Microsoft HoloLens head-mounted display;	MRI	No image-to-patient registration; the hologram	No

Included record	Software or segmentation platform	Display or hardware	Source imaging modality	Registration or alignment method	Quantitative accuracy reported
	of-interest method; separate segmentation platform NR	hologram displayed adjacent to the surgical monitor		was scaled and rotated interactively and related visually to the laparoscopic view	
Torabinia et al. 2022 [20]	Materialise Mimics Research 21.0 for segmentation; Geomagic Wrap for mesh processing; SolidWorks for assembly; Microsoft 3D Viewer for display	Microsoft HoloLens 2, compared against a two-dimensional monitor	MRI — isotropic 0.5 mm T1, fat-saturated T1, and T2 series	Eye-tracking calibration followed by manual orientation of the hologram relative to the laparoscopic view; no automated model-to-specimen registration	No — evaluation was qualitative
Comptour et al. 2025 [21]	NR	Laparoscopic monitor overlay	Preoperative MRI	NR	No
Collins et al. 2021 [22]	NR (custom system)	Monocular laparoscope with monitor overlay; no optical tracker or head-mounted display required	Preoperative MRI or CT	Markerless registration with real-time tracking of the mobile uterus from monocular laparoscopic video	NR
François et al. 2020 [23]	NR (deep-learning occluding-contour detection)	Laparoscopic monitor overlay; touch input for manual contour marking	Laparoscopic video (3,818 labelled images from 10 procedures)	Automated detection of uterine occluding contours used to constrain registration of the preoperative model, which was then tracked for live augmentation	Yes — registration reprojection error and surgeon interaction time reported
Prokopetc et al. 2015 [24]	NR	NR	Laparoscopic images (n = 95)	Automated detection of the uterus and fallopian tube–uterus junctions as registration landmarks	NR
Sato et al. 2019 [17]	OpenCV	NR	Laparoscopic video (19 cases)	Image matching and panoramic reconstruction; ureter detection reported by the authors as unsatisfactory	NR
Madad Zadeh et al. 2023 [25]	NR (SurgAI3.8K dataset)	Not applicable	Laparoscopic images (3,800)	Not applicable — dataset and annotation study	Not applicable
Netter et al. 2025 [7]	YOLOv5	Not applicable	Laparoscopic video from 112 patients	Not applicable — lesion detection, no registration component	Yes — class-specific F1 scores reported, ranging from 0.63 for deep endometriosis to 0.94 for superficial black lesions
Leibetseder et al. 2022 [8]	Faster R-CNN and Mask R-CNN	Not applicable	Laparoscopic video dataset	Not applicable — lesion detection and localization	Yes — detection and segmentation performance reported

Included record	Software or segmentation platform	Display or hardware	Source imaging modality	Registration or alignment method	Quantitative accuracy reported
Flaxman et al. 2024 [26]	NR	Physical 3D-printed model; printer and material NR	MRI	Not applicable — preoperative physical model, no intraoperative registration	No
Li et al. 2026 [27]	NR — the report refers to a supplementary protocol for technical specifications but does not name the platform in the article text	Physical 3D-printed model; printer and material NR	High-resolution MRI	Not applicable — preoperative physical model, no intraoperative registration	No
Martel et al. 2026 [9]	VP Planning	Virtual-reality display; device NR	T2-weighted three-dimensional axial pelvic MRI	Not applicable — preoperative virtual model	No
Borghese et al. 2022 [10]	D2P (DICOM-to-PRINT, 3D Systems); semi-automatic multi-slice interpolation and threshold segmentation	On-screen virtual model; head-mounted display NR	MRI — sagittal and oblique axial T2; coronal T1 urographic phase in selected cases for ureteric reconstruction	Not applicable — preoperative virtual model; concordance with operative findings assessed qualitatively	No — surgeon-rated concordance only
Zhang et al. 2026 [11]	NR	NR	Pelvic MRI	Not applicable — preoperative virtual model	No

Abbreviations: CT, computed tomography; MRI, magnetic resonance imaging; NR, not reported; R-CNN, region-based convolutional neural network; YOLO, You Only Look Once; 3D, three-dimensional.

Authors' note on charting policy. Several of the included records originate from research groups whose software platforms are identifiable from other publications. Those platform names have deliberately not been entered here, because doing so would attribute to a record information that the record itself does not state. Readers seeking implementation detail beyond what is charted here should consult the primary reports directly.
